# P2X7R antagonism suppresses long-lasting brain hyperexcitability following traumatic brain injury in mice

**DOI:** 10.7150/thno.97254

**Published:** 2025-01-27

**Authors:** Mariana Alves, Laura de Diego-Garcia, Gloria Vegliante, Oscar Moreno, Beatriz Gil, Pedro Ramos-Cabrer, Meghma Mitra, Ana Fernandez Martin, Aida Menéndez-Méndez, Yitao Wang, Nathan Ryzewski Strogulski, Meng-Juan Sun, Ciara Melia, Giorgia Conte, Sandra Plaza-García, Igor Khalin, Xinchen Teng, Nikolaus Plesnila, Bert Klebl, Klaus Dinkel, Michael Hamacher, Anindya Bhattacharya, Marc Ceusters, James Palmer, David J. Loane, Jordi Llop, David C. Henshall, Tobias Engel

**Affiliations:** 1 Department of Physiology & Medical Physics, RCSI University of Medicine & Health Sciences, Dublin D02 YN77, Ireland.; 2 Department of Optometry, Faculty of Optics and Optometry, Universidad Complutense de Madrid, Avda. Arcos de Jalon 118, 28040 Madrid, Spain.; 3 School of Biochemistry and Immunology, Trinity Biomedical Sciences Institute, Trinity College, Dublin, Ireland.; 4 CIC biomaGUNE, Basque research and Technology Alliance (BRTA), P° Miramon 182, 20014 San Sebastian, Gipuzkoa, Spain.; 5 Ikerbasque Basque Foundation for Science, Bilbao, Spain.; 6 Department of Medicine, Faculty of Biomedical Sciences and Health, Universidad Europea de Madrid, C. Tajo, s/n, 28670 Villaviciosa de Odón, Madrid, Spain.; 7 College of Pharmaceutical Sciences, Soochow University, Suzhou, Jiangsu 215123, China.; 8 Institute for Stroke and Dementia Research (ISD), LMU University Hospital, Ludwig-Maximilians-University (LMU) Munich, 81377 Munich, Germany.; 9 Munich Cluster for Systems Neurology (SyNergy), 81377 Munich, Germany.; 10 Normandie University, UNICAEN, INSERM UMR-S U1237, Physiopathology and Imaging of Neurological Disorders (PhIND), GIP Cyceron, Institute Blood and Brain @ Caen-Normandie (BB@C), Caen, France.; 11 KHAN Technology Transfer Fund I GmbH & Co. KG, Otto-Hahn-Straße 15, 44227 Dortmund, Germany.; 12 Lead Discovery Center GmbH, Otto-Hahn-Straße 15, 44227 Dortmund, Germany.; 13 Affectis Pharmaceuticals AG, Otto-Hahn-Straße 15, 44227 Dortmund, Germany.; 14 Janssen Research and Development LLC, San Diego, California, USA.; 15 Janssen Pharmaceutica NV, Beerse, Belgium.; 16 The Marc Ceusters Company, BV, Diest, Belgium.; 17 FutureNeuro Research Ireland Centre for Translational Brain Science, RCSI University of Medicine and Health Sciences, Dublin D02 YN77, Ireland.

**Keywords:** Post-traumatic Epilepsy, Traumatic Brain Injury, Epileptogenesis, P2X7 Receptor, Positron Emission Tomography, Magnetic Resonance Imaging, Microglia

## Abstract

**Purpose:** Post-traumatic epilepsy (PTE) is one of the most common life-quality reducing consequences of traumatic brain injury (TBI). However, to date there are no pharmacological approaches to predict or to prevent the development of PTE. The P2X7 receptor (P2X7R) is a cationic ATP-dependent membrane channel that is expressed throughout the brain. While increasing evidence suggests a role for the P2X7R during seizures and epilepsy, it is unclear if changes in P2X7R expression can predict TBI-induced epilepsy development, and whether P2X7R antagonism can protect against long-lasting brain hyperexcitability caused by TBI.

**Methods:** TBI was induced in adult male mice using the controlled cortical impact model (CCI). To test the anti-epileptogenic effects of P2X7R antagonism, mice were treated with brain-penetrant P2X7R antagonists JNJ-54175446 (30 mg/kg) or AFC-5128 (30 mg/kg) for 7 days post-CCI. The cell-type specific effects of P2X7Rs on TBI-induced hyperexcitability were analyzed in mice lacking exon 2 of the *P2rx7* gene selectively in microglia (*P2rx7*:*Cx3cr1*-Cre). Static positron emission tomography (PET) via an intravenous injection of the P2X7R radioligand ^18^F-JNJ-64413739 and magnetic resonance imaging (MRI) were conducted twice during the first- and third-week post-injury.

**Results:** Following TBI, while there were no obvious changes in P2X7R protein levels in the ipsilateral hippocampus post-injury, there was a delayed increase in P2X7R protein levels in the ipsilateral cortex at 3 months post-injury. Treatment with P2X7R antagonists shortly after TBI reduced long-lasting brain hyperexcitability, reduced cortical contusion volume, and normalized injury-induced hyperactivity to control sham-levels at 3 weeks post-TBI. Notably, mice lacking *P2rx7* in microglia had an increased seizure threshold after TBI, suggesting that P2X7R contributed to brain hyperexcitability via its effects on microglia. Finally, P2X7R radioligand uptake after TBI correlated with seizure threshold at 3 weeks post-injury.

**Conclusions:** Our results demonstrate the antiepileptogenic potential of P2X7R antagonism to prevent TBI-induced epilepsy and indicate that P2X7R-based PET imaging may be a useful diagnostic tool to identify people at risk of developing PTE.

## Introduction

Traumatic brain injury (TBI) affects ~70 million people worldwide and is one of the main causes of acquired epilepsy, resulting in an estimated 20% of symptomatic epilepsies 1, 2. Post-traumatic epilepsy (PTE) is a devastating long-term complication associated with TBI and has been shown to affect between 13 - 50% of patients with severe TBI depending on the population under investigation and time points studied 3, 4. Anti-seizure medications (ASMs) can successfully stop seizures in ~70% of epilepsy patients, but there is a large population of patients who are resistant to ASM treatment. Moreover, ASMs frequently cause unwanted side-effects (*e.g.,* fatigue, irritability) and they target symptoms without a significant impact on disease progression 5, 6. Critically, PTE has a particularly high incidence of pharmacoresistance 7, and there are no currently available drugs to prevent PTE development or validated biomarkers that predict its onset 8-11.

A number of pathological processes occur during the seizure-free latent period in the brain following a TBI, including neuronal cell death, oxidative stress, altered neurogenesis and neuroplasticity, and neuroinflammation 12. Neuroinflammation, which involves enhanced glial reactivity including activated microglia and astrocytes, the release of pro- and anti-inflammatory immunomodulators, disruption of the blood-brain barrier (BBB), and infiltration of peripheral leukocytes and lymphocytes into the injured brain, has been suggested as one of the main contributors to PTE 13-15.

ATP functions as an important damage-associated molecular pattern (DAMP) 16 driving inflammatory processes in the brain 17. Extracellular ATP increases rapidly under pathological conditions (*e.g.,* seizures), activating ionotropic P2X receptors (P2XRs), a family of cation-selective membrane channels permeable to Na^+^, K^+^ and Ca^2+^, on neurons and glial cells 18, 19. Among the P2XR family, the P2X7 receptor (P2X7R) has unique structural and functional characteristics, most notably, a relatively low affinity for ATP (EC50 ≥ 100 μM) 20, suggesting that P2X7R activation occurs mainly under pathological conditions of high ATP release. Antagonism of the P2X7R may avoid, therefore, interference with normal physiological actions of ATP and have a more favorable side-effect profile. The P2X7R mediates a wide array of pathological functions in the injured brain, including pro-inflammatory cytokine signaling (*e.g.* Interleukin-1β (IL-1β)) that may be related to its high expression in microglia 21, 22. In addition, P2X7R activation is linked to other processes during epileptogenesis, including cell death, synaptic plasticity, disruption of the BBB, and neurotransmitter release 23. A causal role for P2X7R-dependent signaling during the generation of seizures and epilepsy has been documented 24. P2X7R expression has been shown to increase in the injured brain following status epilepticus (SE) in animal models and patients with epilepsy 25. Notably, P2X7R antagonism in rodents reduces seizure severity and brain damage during SE and decreases seizure frequency and neuroinflammation in epilepsy 26, 27. Moreover, recent data show that increased P2X7R expression in mice contributes to unresponsiveness to ASMs targeting neurotransmission (*e.g*., lorazepam, carbamazepine) during SE 28.

While studies have investigated the potential of P2X7R antagonism as treatment for TBI-induced pathologies, these have been restricted to its effects on acute brain injury and neurobehavioral outcomes 29, 30. Specifically, blocking P2X7R decreases TBI-induced microglial activation and associated neurodegeneration, as well as neurobehavior deficits such as hyperactivity and cognitive dysfunction 29-32. Timing of treatment seemed, however, to be critical, with too early P2X7R-based interventions during TBI leading to adverse outcomes 33. Whether P2X7R antagonism affects TBI-induced brain hyperexcitability has not been investigated to date.

The uncertainty and complexity of epilepsy development underpin the need to develop biomarkers that can accurately predict the progression of PTE. TBI-induced epilepsy occurs following a latent period of months to years 8, which represents the potential treatment window. Moreover, the identification of mechanistic biomarkers of epileptogenesis may provide better insights into the process of disease development, help identify patients who would benefit from a specific treatment and provide quantitative parameters to assess therapeutic efficacy. Positron emission tomography (PET) is a powerful, non-invasive, and clinically established tool for the identification of disease-specific biomarkers. Importantly, recent studies have shown P2X7R-based PET tracer uptake to correlate with seizure-induced pathology 34, 35.

Here, using a controlled cortical impact (CCI) mouse model, we show brain region-specific and temporal P2X7R expression changes following TBI. Pharmacological antagonism of the P2X7R initiated after TBI reduced long-lasting brain hyperexcitability and cortical tissue damage, most likely by blocking microglial-mediated processes. Finally, we show that P2X7R PET radioligand uptake negatively correlates with seizure threshold post-TBI, indicating that P2X7R activation may increase the susceptibility to develop PTE in the chronic phase of recovery. Thus, our data identify P2X7R antagonism as a potential novel treatment to protect against TBI-induced epilepsy development.

## Material and Methods

### Animals

All animal experiments were performed in accordance with the principles of the European Communities Council Directive (2010/63/EU). Procedures were reviewed and approved by the Research Ethics Committee of the Royal College of Surgeons in Ireland (RCSI) (REC202012013 and REC1062) and Health Products Regulatory Authority (HPRA) (AE19127/P070 and AE19127/P027). For experiments conducted at CIC biomaGUNE, procedures were reviewed and approved by the internal Ethical Committee and approved by the local authorities (Diputación Foral de Gipuzkoa; PRO-AE-SS-208). All animals were treated according to European standards and regulations for animal experiments and all efforts were made to minimize animal suffering and reduce the numbers of animals used. All mice used in our experiments were 10 - 14 weeks old males, with a weight range between 25 - 35 g. The following strains were used: FVB/NJ wild-type (wt) mice for all experiments except for experiments in Figure 2E-F where we used mP2X7-EGFP BAC transgenic mice (FVB/N-Tg(RP24- 114E20-P2X7/StrepHisEGFP)) that overexpress a P2X7 protein that is C-terminally fused to the enhanced green fluorescent green protein (EGFP) and is expressed under the control of the BAC-derived *P2rx7* promoter 22 (P2X7-EGFP mice). For experiments in Figure 5, we crossed a tamoxifen-inducible microglia Cre line B6.129P2(Cg)-Cx3cr1tm2.1(cre/ERT2)Litt/WganJ (*Cx3cr1*) (JAX stock #021160) with mice where the murine exon 2 of the *P2rx7* gene is flanked with loxP sites *(P2rx7^fl/fl^*) (generated by the European Conditional Mouse Mutagenesis (EUCOMM) Program [P2rx7tm1a(EUCOMM)Wtsi]) to obtain conditional mice with a *P2rx7* deletion in microglia (*P2rx7^-/-^*-M). *P2rx7^fl/fl^* mice without Cre (but treated with tamoxifen) were used as control. Cell-specific tamoxifen-inducible knockout mice were on the C57BL/6J background. All animals were housed in a controlled biomedical facility using Tecniplast conventional cages (Ref. 1284L EUROSTANDARD TYPE II L) and Lignocel BK8/15-25, premium hygienic animal bedding (D0764P00Z) with 2 - 5 mice per cage on a 12-hour light/dark cycle at 22.0 ± 1.0 ºC and humidity of 40 - 60% with food and water provided *ad libitum*. For each cage, enrichment was provided in the form of nesting material (irradiated Bed-R'Nest Brown, Dates and, Item code CS1BEB), PVC tubes and red polycarbonate mouse houses. All *in vivo* studies were carried out during the light phase of the cycle.

### Controlled Cortical Impact (CCI) model of TBI

**Figure 1:**
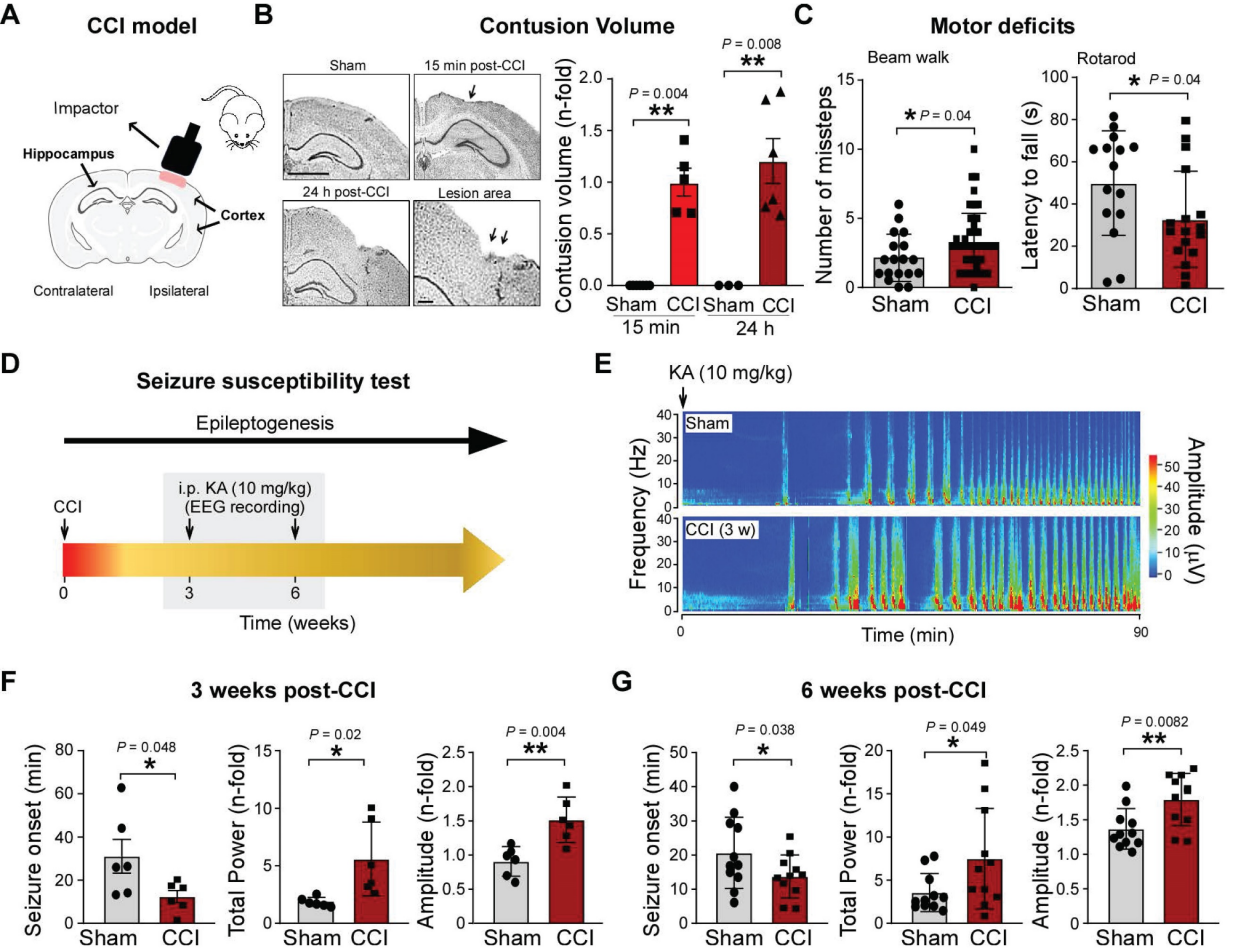
**CCI leads to long-lasting brain hyperexcitability. (A)** Schematic showing the CCI model in mice. **(B)** Representative images and graphs showing contusion volume in the ipsilateral cortex 15 min and 24 h post-CCI (n = 7 (sham-15min), 3 (sham-24h), 5 (15 min post-CCI) and 6 (24 h post-CCI)). **(C)** Foot faults assessed by the Beam walk (n = 18 (sham) and 63 (CCI)) and latency to fall using the Rotarod (n = 15 (sham) and 17 (CCI)) 24 h post-CCI. Unpaired Student's t-test.** (D)** Experimental approach to test for long-lasting brain hyperexcitability post-CCI. Mice were administered KA (10 mg/kg, i.p.) 3 and 6 weeks post-CCI and EEG was recorded for 90 min post-KA injection. **(E)** Representative EEG recording represented via heat map from mice (sham- and CCI) administered i.p. KA 3 weeks post-injury. **(F)** Graphs showing seizure onset, total power and amplitude of EEG recording from mice administered i.p. KA 3 weeks post-CCI (n = 6 per group). Unpaired Student's t-test. **(G)** Graphs showing EEG total power and amplitude of mice (sham- and CCI) administered i.p. KA 6 weeks post-CCI (n = 11 per group). Unpaired Student's t-test. Data are shown as mean ± SD. Created with BioRender.com. *P < 0.05; **P < 0.01.

CCI was induced via a custom-made device consisting of a pneumatic impactor with a 3.0 mm diameter tip 36. Briefly, mice were anesthetized with isoflurane (5% induction, 1 - 2% maintenance) and core body temperature was maintained at 37°C using a heating pad. Before surgery, mice were administered with buprenorphine (0.05 mg/kg, i.p.). The head was mounted in a stereotaxic frame, a 10 mm midline incision was made over the skull and the skin and fascia were reflected. A 4 x 4 mm craniotomy was made on the right parietal bone, between bregma and lambda (Figure 1A). The mouse head was rotated 3° from the horizontal to the left (lateral impact). The impounder tip of the injury device was extended to its full stroke distance, positioned to the intact surface of the exposed dura mater, and reset to impact the cortical surface. CCI was induced using an impactor velocity of 8 m/s, deformation depth of 1 mm, and a dwell time of 150 ms 36. After injury, the cranial window was immediately closed with the bone fragment put back in place with tissue glue (World Precision Instrument, UK; Vetbond). The skin incision was closed with interrupted 5-0 silk sutures, anesthesia was terminated, and the animal was placed into a heated incubator to maintain normal core temperature for 45 min post-injury before being returned to their home cage. Sham mice underwent the same procedure as CCI mice (anesthesia and craniotomy) except for the impact.

### Analysis of EEG and behavior changes during seizures

Changes in electroencephalogram (EEG) activity were recorded from cortical implanted electrodes. Briefly, during stereotaxic procedures, mice were anesthetized using isoflurane (5% induction, 1 - 2% maintenance) and maintained normothermic by means of a feedback-controlled heat blanket (Harvard Apparatus Ltd, Kent, UK). The depth of the anaesthesia was frequently tested by checking the plantar nociception or corneal reflex. Additionally, to minimize pain during and post-surgery, mice were treated with buprenorphine (0.05 mg/kg, i.p.), and EMLA cream (Aspen Pharma, UK) which was applied to head wounds and ear bars. Once fully anesthetized, mice were placed in a stereotaxic frame and a midline scalp incision was performed to expose the skull. Then, two cortical electrodes, one on top of the undamaged, contralateral hippocampus and the reference electrode on top of the frontal cortex, were fixed in place with dental cement. EEG was recorded for 10 min before and for 90 min following an administration of kainic acid (KA) (10 mg/kg, i.p.). To analyze seizure severity, data were uploaded onto Labchart7 software (AD Instruments) as before 37. EEG total power (µV^2^) is a function of EEG amplitude over time and was analyzed by integrating frequency bands from 0 - 100 Hz. Power spectral density heat maps were generated within LabChart7 (spectral view), with the frequency domain filtered from 0 - 40 Hz and the amplitude domain filtered from 0 - 50 mV. Data is presented as n-fold of baseline recordings prior to injection of KA, if not indicated otherwise. Seizure onset was analyzed offline. Seizure onset was defined as first seizure burst detectable on the EEG consisting of high amplitude (> twice baseline) high frequency polyspiking of a minimum of 5 s in duration. Behavioral seizures were scored according to a modified Racine Scale, as reported previously 38. Score 1, immobility and freezing; score 2, forelimb and or tail extension, rigid posture; score 3, repetitive movements, head bobbing; score 4, rearing and falling; score 5, continuous rearing and falling; score 6, severe tonic-clonic seizures. Mice were scored every 5 min for 90 min after KA injection. The highest score attained during each 5 min period was recorded by an observer blinded to treatment. The final score for each mouse was the average of each score over the 90 min recording period.

### Drug administration

To identify the optimal drug treatment regimen we chose two different treatment windows, one starting at day 1 post-CCI (*i.e*., acutely) and the other starting at day 7 post-CCI (*i.e*., the peak in post-traumatic neuroinflammation 39). To block P2X7Rs, we used two brain penetrant P2X7R antagonists that have been shown to produce robust seizure-suppressive effects in other central nervous system (CNS) injury models 28, 40, 41. JNJ-54175446 (30 mg/kg) 41 or vehicle (30% hydroxypropyl-methyl cellulose [Sigma-Aldrich]) were delivered via oral gavage (10 ml/kg) once a day for 7 consecutive days, starting at day 1 to day 7 post-injury (treatment window 1), or from day 7 to day 13 post-injury (treatment window 2). AFC-5128 (30 mg/kg) 40 or vehicle (N,N-dimethylacetamide [DMA, Sigma-Aldrich]) and 45% 2-hydroxypropyl-ß-cyclodextrin (45% ß-CD, Sigma-Aldrich) (final ratio of the vehicle 1:9)] were delivered via intraperitoneal (i.p.) injection (10 ml/kg) once a day, starting from day 7 to day 13 post-injury. The ASM carbamazepine (40 mg/kg, Sigma-Aldrich) or vehicle (45% ß-CD [Sigma-Aldrich]) were delivered via i.p. injection (10 ml/kg) once a day, starting at day 1 to day 7 post-injury, or from day 7 to day 13 post-injury. Carbamazepine is a frequently used drug for patients with epilepsy and this drug has also been tested in TBI patients 42. All mice within the same experimental group received corresponding vehicle treatments. To knock-out P2X7R expression in microglia, tamoxifen (40 mg/kg; prepared in 10% of 100% ethanol and 90% of peanut oil; injection volume = 100 µl) was administered for a period of 7 consecutive days by an i.p. injection as before 43, starting at day 7 until day 13. All mice received the same tamoxifen treatment regime. The tetracycline-type antibiotic minocycline (30 mg/kg, prepared in dH_2_O; Sigma-Aldrich), which has demonstrated anti-inflammatory effects 44, was delivered via an i.p. injection (200 µl) for 7 consecutive days at the same time as P2X7R antagonist treatment (treatment window 2, day 7 - 13) 37.

### Behavioral assessment

*Beam walk*: Motor coordination and balance were assessed by measuring the number of foot faults of the hind paw of a trained mouse walking twice on an elevated, narrow wooden beam (1 cm wide, 1 m long). Mice were trained on the beam walk for 3 days prior to surgery in three habituation trials. Performance was assessed 24 h post-CCI and was scored as the sum of the number of foot faults during the two tests (best score 0).

*Open field test*: Mice were placed in an arena made of four transparent Plexiglas walls (30 x 30 x 30 cm) and recorded for 10 min using an overhead Logitech Webcam C270 (720p) 45. Several parameters, including the total distance travelled, total time mobile, number of crossings, time spent in center and corner, and average speed, were analyzed automatically with ANY-maze video tracking system (Version 6.32).

*Rotarod*: The Rotarod was set with a starting speed of 4 rpm and acceleration rate 10 rpm during 10 min of test. Before each test, mice were trained 2 days prior to surgery in two habituation trials (three consecutive trials/day). The test was performed 24 h post-CCI and the latency to fall was scored as the average time obtained in the three trials.

### RNA extraction and qPCR

Following cervical dislocation and subsequent perfusion of mice with ice-cold phosphate-buffered saline (PBS), hippocampi and cortex were dissected and put on dry ice and stored at -80 ºC for further processing. RNA extraction was performed using the Trizol method, as described before 37. Quantity and quality of RNA was measured using a Nanodrop Spectrophotometer (Thermo Scientific, Rockford, IL, U.S.A). Samples with a 260/280 ratio between 1.8 - 2.0 were considered acceptable. 500 ng of total RNA was used to produce complementary DNA (cDNA) by reverse transcription using SuperScript III reverse transcriptase enzyme (Invitrogen, CA, U.S.A) primed with 50 pmol of random hexamers (Sigma, Dublin, Ireland). Quantitative real-time polymerase chain reaction (qPCR) was performed using the QuantiTech SYBR Green kit (Qiagen Ltd, Hilden, Germany) and the LightCycler 1.5 (Roche Diagnostics, GmbH, Mannheim, Germany). Each reaction tube contained 2 μl cDNA sample, 10 μl SyBR green Quantitect Reagent (Qiagen Ltd, Hilden, Germany), 1.25 μM primer pair (Sigma, Dublin, Ireland) and RNAse free water (Invitrogen, CA, U.S.A) to a final volume of 20 μl. Using LightCycler 1.5 software, data were analyzed and normalized to *β-actin* mRNA levels. Primers used (Sigma, Dublin, Ireland): *p2rx7* forward: actggcaggtgtgtgttccata, reverse: ttggcaagatgtttctcgtg; and *β-actin* forward: gggtgtgatggtgggaatgg, reverse: ggttggccttagggttcagg.

### Western blotting

Western blot analysis was performed as described previously 37. Lysis buffer (100 mM NaCl, 50 mM NaF, 1% Triton X-100, 5 mM EDTA pH 8, 20 mM HEPES pH 7.4) containing a cocktail of phosphatase and protease inhibitors (Sigma-Aldrich) was used to homogenize ipsilateral cortex or hippocampal brain tissues and to extract proteins, which were quantified using a Tecan plate reader at 560 nm. 30 µg of protein samples were loaded onto an acrylamide gel and separated by 10 - 12% sodium dodecyl sulphate-polyacrylamide gel electrophoresis (SDS-PAGE). Following electrophoresis, proteins were transferred to a nitrocellulose membrane (GE Health Care, Illinois, USA) and immunoblotted with the following primary antibodies: P2X7R (1:500, rabbit IgG, directed against epitope AA 363 to 595 of mouse P2X7Rs, Synaptic System, Göttingen, Germany; Cat #: 177003), P2X7R intracellular (1:400, rabbit IgG; Alomone Labs, directed against epitope AA 576-595 of rat P2X7R, Jerusalem, Israel; Cat #: APR-004), IBA-1 (1:400 rabbit IgG; WAKO chemicals GMBH, Neuss, Germany; Cat #: 019-19741), GFAP (1:1000, mouse IgG; Sigma-Aldrich, Dublin, Ireland; Cat # G3893) and P2X4R (1:400, rabbit IgG; Alomone Labs, Jerusalem, Israel Cat # APR-002) all prepared in 5% milk-tris buffered saline-tween (TBST). Membranes were incubated with horseradish peroxidase (HRP)-conjugated goat anti-rabbit or anti-mouse (1:5000, Millipore, Massachusetts, USA; Cat #: AP132P) in 5% milk-TBST. Protein bands were visualized using Fujifilm LAS-4000 system with chemiluminescence (Imombilon western HRP substrate, Merck Millipore, Massachusetts, USA) followed by analysis using Alpha-EaseFC4.0 software. Protein quantity was normalized to the loading control β-ACTIN (1:1000 prepared in 5% milk-TBST; anti-mouse; Sigma-Aldrich, Arklow, Ireland; Cat # A5316) or α-TUBULIN (1:1000 prepared in 5% milk-TBST; anti-mouse; Sigma-Aldrich, Arklow, Ireland; Cat # T5168).

### Histopathology

To determine the contusion volume, mice were sacrificed 15 min, 24 h and 21 days post- injury by cervical dislocation, and brains were carefully frozen in powdered dry ice and stored at -80°C until use. Fourteen sequential 10 µm coronal sections (starting 500 µm behind olfactory bulb) were cut at 500 µm intervals on a cryostat (Leica, Germany) and stained with Cresyl Violet. Briefly, sections were incubated for 10 min with 70% ethanol, then 20 min with 1% Cresyl Violet solution (Sigma-Aldrich, C5042). Sections were then washed once in distilled water, twice in 70% ethanol, 96% ethanol and 100% ethanol followed by 2 min in 100% isopropanol. Sections were cleared with xylol I for 10 min followed by xylol II for 10 min, and mounted with DPX mounting medium (Vector Laboratories, Burlingame, CA, USA). Images (2.5x) were acquired using a light microscope (Leica), and the extent of traumatic brain damage in the ipsilateral hemisphere was manually outlined by researcher blinded to treatments using the ImageJ software (Rasband, W.S., ImageJ, U. S. National Institutes of Health, Bethesda, Maryland, USA, https://imagej. nih. gov/ ij/, 1997-2018). The contusion volume (V) was calculated by integration of the injured area according to the following formula: V = d * [(A1)/2 + A2 + A3…+ (An)/2], whereby 'd' is the distance between slices in mm (*i.e*., 0.51 mm: slice thickness + interslice gap), and 'A' is the injured area in mm^2^ calculated for each of the 14 (n) coronal sections/mouse 46. Data are represented as n-fold to control.

### Immunofluorescence

Immunofluorescence was carried out as described previously 45. P2X7-EGFP reporter mice were transcardially perfused with 4% paraformaldehyde (PFA) and post-fixed for an additional 24 h. Brains were then transferred to PBS and immersed into 4% agarose before sectioning. 30 µm sagittal sections were cut using the VT1000S vibratome (Leica Biosystems, Wetzlar, Germany) and sections were stored at -20 ºC in glycol. For immunofluorescence staining, tissue sections were incubated with 0.1% triton/PBS and 1 M glycine, followed by blocking with 1% BSA-PBS for 45 min. Sections were then incubated with the primary antibody GFP (1:400; rabbit IgG; Thermo Fisher Diagnostics, Inchinnan, UK; Cat # A11122) overnight, which was followed by IBA-1 (1:400: goat IgG; Abcam, Cambridge, UK; Cat # AB5076), Olig2 (1:400; mouse IgG, Millipore, Dublin, Ireland; Cat # Cat # MABN50), GFAP (1:400, mouse IgG; Sigma-Aldrich, Arklow, Ireland, Cat # G3893) or synaptophysin, (1:400; mouse IgG; Abcam, Cambridge, UK, cat # AB8049) the next day at room temperature for 2 h. After washing in PBS, tissue was incubated with fluorescent secondary antibodies, AlexaFluor488 or AlexaFluor568 (1:400; Invitrogen, Dublin, Ireland). This was followed by washing in PBS and a 5 min incubation with DAPI (1:500; Sigma-Aldrich, Arklow, Ireland). *FluorSave*™ (Merck Millipore, Massachusetts, USA; Cat # 345789) was used to cover the tissue and confocal images were taken with a Zeiss 710 LSM NLO confocal using a 40x oil immersion objective and ZEN 2010B SP1 software. Cell counts were taken as the average of two images (40x), corresponding to an area of 0.045 mm^2^, per subfield. Cell counts were divided by area, to determine an approximate value for cell density in each brain region. The hippocampus was further subdivided into three distinct areas: cornu ammonis 1 (CA1), cornu ammonis 3 (CA3) and dentate gyrus (DG). Final counts are represented as cell number per mm^2^.

To determine the impact of P2X7R antagonist treatment on microglia number post-CCI, sections were fixed in 4% PFA which was followed by permeabilization with 3% Triton and blocking with 5% goat serum. Sections were then incubated overnight with the specific cell type marker IBA-1 (1:400 prepared in 5% goat serum; rabbit IgG; WAKO chemicals GMBH, Neuss, Germany; Cat #: 019-19741). After rinsing with PBS, slices were incubated with secondary antibodies conjugated to AlexaFluor 568 (1:400 prepared in 5% goat serum; rabbit IgG, BioSciences, Dublin, Ireland; Cat #: A-11011) for 2 h at room temperature, followed by DAPI incubation. Sections were mounted using *FluorSave*™ (Merck Millipore, Massachusetts, USA; Cat # 345789) and three images from each hippocampal subfield, (CA1, CA3 and DG), along with two from two cortical adjacent areas, located below the area of impact area, were obtained using a 20x lens in the Nikon 2000s epifluorescence microscope. The number of cells was counted by a person blinded to treatment, and calculated by taking an average of the two pictures obtained.

### Radiochemistry

According to previous reports, the radioligand ^18^F-JNJ-64413739 can be used to provide insight into P2X7R expression in the brain by means of PET imaging 47. The radioligand was prepared at CIC biomaGUNE (San Sebastian, Spain) following a previously published method 35 (see Supplementary Material for experimental details).

### *In vivo* PET-CT studies

Positron emission tomography (PET) and computerized tomography (CT) imaging studies were conducted using β- and X-cube microsystems, respectively (Molecubes, Belgium) at two time-points post-CCI/sham (1^st^ imaging: 7 days post-injury; 2^nd^ imaging: 17 days post-injury) for each animal. Anesthesia was induced with 3 - 4% isoflurane in pure oxygen and maintained during imaging studies with 1 - 1.5% isoflurane in pure oxygen. Anesthetized CCI (n = 10) and sham mice (n = 5) were injected intravenously (i.v.) with ^18^F-JNJ-64413739 (2.3 ± 0.4 MBq, 30 µl in 10% ethanol) via one of the lateral tail veins. Thirty minutes after radioligand administration, animals were positioned in the PET scanner and 30 min static images were acquired in one bed position, with animals centered in the middle of the field of view. Scan times were corrected for the delay in PET experiment initialization. A 5 min CT scan (X-Ray energy: 40 kV; intensity: 140 μA) was acquired immediately after each PET acquisition. PET images were reconstructed by OSEM-3D iterative algorithm and analyzed using π-MOD image analysis software (π-MOD Technologies Ltd, Zurich, Switzerland). Volumes of interest (VOIs) were delineated in different brain regions (whole brain and ipsi- and contralateral cerebral cortex, hippocampus, striatum, cerebellum, thalamus, and amygdala) using the M. Mirrione-T2 magnetic resonance imaging template. The concentration of radioactivity in the different brain regions was determined and normalized to the injected radioactivity and animal weight to be expressed as Standardized Uptake Value (SUV).

### Magnetic resonance imaging

The same cohort of sham and TBI mice underwent MRI between days 8 - 11 (1^st^ imaging) and days 16 - 21 (2^nd^ imaging) post-injury, using a 117/16 USR Bruker Biospec system (Bruker Biospin GmbH, Ettlinglen, Germany) interfaced to an advance III console and operating ParaVision 6.1 software (Bruker Biospin). Images were acquired using a 7 ​cm inner diameter volumetric coil for radiofrequency transmission (TX) from Bruker, and a single-channel mouse brain surface coil (Neos Biotec, Pamplona, Spain) for detection (RX). Acquisition was conducted using Burke's ParaVision 7.1 software. All imaging experiments were conducted under isoflurane anesthesia (4% for induction and 1- 2% for maintenance) carried by a 1.5 l/min flow of a 50/50 mixture of O_2_/N_2_ gas. Animal temperature (37 ± 0.1 °C) and respiration rate were continuously monitored using a SAII 1030 monitoring system from Small Animal Instruments (Stony Brook, NY, USA), and corrected by a feedback-controlled hot air current provided by the SAII 1030 system. The imaging protocol included a scout imaging for correct positioning of the animal followed by a T2 weighted image acquired using a RARE (Rapid Acquisition with Relaxation Enhancement) pulse sequence with the following imaging parameters: RARE factor = 4; Effective eco time TE = 36 ms (TE = 18 ms); Repetition time TR = 6 s; N_AV_ = 2 averages. K space zero filling factor of 1.34 (read-out) and 1.34 (phase encoding); image matrix of 256 x 256 points, covering a field of view of FOV = 15 x 15 mm^2^, giving an in-plane resolution of 59 x 59 µm^2^. The whole brain was covered by acquiring 18 slices of 0.5 mm of thickness (9 mm long imaging slab). Images were acquired with a sweep width of 66 kHz (257.8 Hz/pixel). For image processing, raw Bruker images were transferred to the NIH software Image-J and inspected by an MRI expert with 20 years of experience in preclinical MRI (P. R-C) blinded to the study. Left (contralateral) and right (ipsilateral) cortical volumes were measured by manually drawn regions of interest (ROIs) and the ratio Vipsi/Vcontra (expressed in %) was calculated for all animals (n = 15) at both time points measured.

### Statistical analysis

Statistical analysis of data was carried out using GraphPad Prism 5 and STATVIEW software (SAS Institute, Cary, NC, U.S.A). Data are presented as standard deviation (SD) if not indicated otherwise in Figure legends. One-way ANOVA parametric statistics with *post hoc* Fisher's protected least significant difference test was used to determine statistical differences between three or more groups. Unpaired Student's t-test was used for two-group comparison. Two-way ANOVA was used for repeated measures between groups where a series of measurements have been taken from the same mouse at different time-points. Correlations between variables were assessed using Spearman's Rank correlation coefficient. Detailed *P* values are given within Figures or Figure legends. To verify the existence of outliers, we employed the ROUT test (Q = 0.1%), a robust statistical method for outlier detection. This test helps identify data points that are significantly different from the rest of the dataset, ensuring the accuracy of our analysis. Significance was accepted at *P < 0.05.

## Results

### Long-lasting brain hyperexcitability after TBI

The CCI model (Figure 1A) is routinely used to study the pathophysiology of TBI-induced epilepsy with mice subjected to CCI typically developing epilepsy after a seizure-free latent period of 2 - 3 months 48. However, before analyzing the role of P2X7R during PTE, we first carefully assessed whether our model recapitulates pathological changes occurring following CCI, as reported previously 48.

The progressive cortical damage induced by CCI was assessed at 15 min and 24 h post-CCI by Nissl staining in injured mice (Figure 1B**)**. Of note, damage was similar between the two different background strains used for our studies: FVB/NJ and C57/Bl6 (Figure S1A-B). CCI mice also showed a defect in motor balance and coordination as assessed by the Beam walk test and rotarod 24 h after injury (Figure 1C). To determine whether CCI leads to a lower seizure threshold post-injury, mice were administered KA (10 mg/kg, i.p.) at 3 and 6 weeks post-CCI (or sham). EEG was recorded for 90 min and seizure onset and severity analyzed (Figure 1D). Mice subjected to CCI showed an earlier seizure onset and experienced an approximately 2-fold increase in seizure severity including seizure total power and amplitude when compared to sham mice during a 90 min recording period starting at the time of i.p. KA injection 3 weeks post-CCI (Figure 1E-F). Earlier seizure onset and increased susceptibility to KA were also evident 6 weeks post-TBI (Figure 1G**)**.

### Brain region-specific P2X7R expression changes following TBI

**Figure 2:**
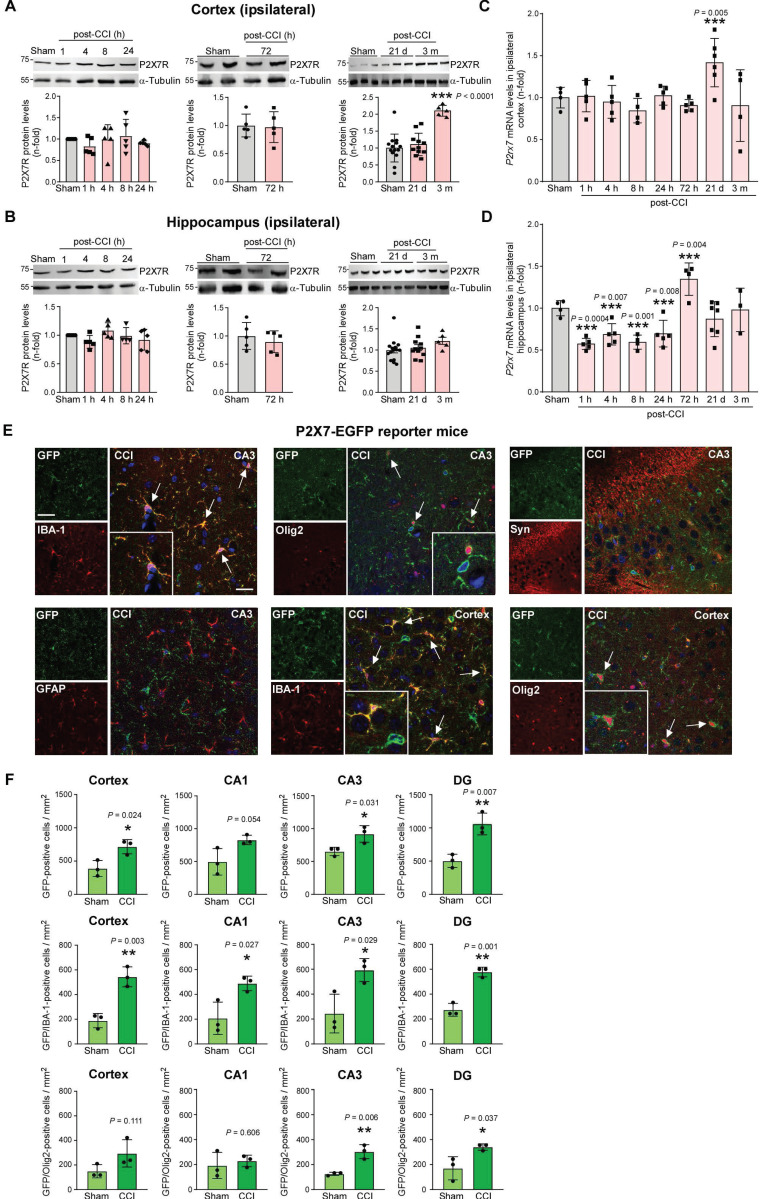
**P2X7R expression post-CCI in the ipsilateral cortex and hippocampus.** Representative Western blots and graphs showing P2X7R protein levels (Synaptic Systems Antibody) in ipsilateral cortical **(A)** and hippocampal **(B)** tissue at different timepoints post-CCI (cortex and hippocampus (n = 5 (24 h and 72 h sham), 5 (post-CCI: 1 h, 4 h, 8 h, 24 h, 72 h), 15 (sham: 21 days and 3 months) and 16 (post-CCI: 21 days and 3 months). One-way ANOVA followed by Fischer's multiple-comparison test. **(C, D)** *P2rx7* transcript levels at different time-points post-CCI in the ipsilateral cortex and hippocampus (Cortex: n = 4 (24 h sham), 5 (post-CCI: 1 h, 4 h, 24 h, 72 h), 4 (post-CCI: 8 h and 3 months) and 6 (post-CCI: 21 days); Hippocampus: n = 4 (24 h sham), 5 (post-CCI: 1 h, 4 h, 24 h), 4 (post-CCI: 8 h and 72 h), 7 (post-CCI: 21 days) and 3 (post-CCI: 3 months)). One-way ANOVA followed by Fischer's multiple-comparison test, CCI *vs* sham mice. **(E)** Representative images showing co-expression of GFP (green) with IBA-1 (red) and Olig2 (red) 21 days post-CCI in the cortex and the hippocampal subfield CA3. No co-expression was observed between GFP (green) and GFAP (red) and synaptophysin (red) at the same time-point post-CCI in CA3. DAPI is shown in blue. Scale bar = 100 µm (small images, left) and 20 µm (merge image, right). **(F)** Quantification of GFP-positive cells in the cortex and all three hippocampal subfields (CA1, CA3 and DG) in P2X7-EGFP reporter mice 3 weeks post-CCI (or sham) and GFP-positive cells co-expressed with the microglia marker IBA-1 and oligodendrocyte marker Olig2 (n = 3 per group). Unpaired Student's t-test. Data are shown as mean ± SD. *P < 0.05; **P < 0.01; ***P < 0.001.

We next investigated P2X7R protein levels at different time-points post-injury, focusing our analysis on the ipsilateral cortex and hippocampus using two different P2X7R antibodies. When analyzing the cortex, we found no significant changes in P2X7R protein levels acutely (1 - 72 h post-CCI). P2X7R expression was, however, increased 3 months post-CCI by approximately 2-fold when compared to control confirmed by both P2X7R antibodies (Figure 2A and Figure S2A, C). We also analyzed the expression of the glial markers IBA-1 (microglia) and GFAP (astrocytes) and the purinergic receptor P2X4R, which is more closely related to the P2X7R regarding its sequence than the other P2XR subtypes 49. While IBA-1 protein levels were significantly increased at 3 months post-CCI, mimicking changes in P2X7R levels, GFAP protein levels were increased acutely (*i.e.*, 1 h, 4 h, 8 h and 24 h post-CCI) and at 3 months post-CCI (Figure S2A, C). Similar to P2X7R protein levels, P2X4R protein levels where higher at 3 months post-CCI when compared to sham (Figure S2A).

When focusing on the hippocampus, while no obvious changes in P2X7R protein levels were observed between sham and CCI-mice post-CCI using the P2X7R antibody directed against epitope AA 363 to 595 of mouse P2X7Rs (Figure 2B), analysis using a second P2X7R antibody directed against epitope AA 576-595 of rat P2X7R showed slightly increased P2X7R protein levels 8 h post-CCI (Figure S2B, C). Moreover, whereas no changes in IBA-1 protein levels were observed at the timepoints analyzed, GFAP was upregulated 21 days post-CCI. No changes in P2X4R protein levels were observed (Figure S2B, C). When analyzing *P2rx7* transcript levels, *P2rx7* mRNA levels seemed to be reduced in the hippocampus while no changes could be observed in the cortex acutely post-CCI. *P2rx7* mRNA levels were, however, increased at 21 days post-CCI in the cortex and 72 h post-CCI in the hippocampus (Figure 2C-D and Figure S2D), suggesting that in addition to *P2rx7* transcription, also post-transcriptional changes may contribute to changes in P2X7R protein levels observed post-CCI.

Using a reporter mouse where P2X7R is expressed under the control of the BAC-derived *P2rx7* promoter and fused to EGFP 22, increase in P2X7R-EGFP expression was evident in microglia and to some extent in oligodendrocytes 3 weeks post-CCI (ipsilateral cortex and hippocampus) (Figure 2E-F and Figures S3). Of note, EGFP-P2X7R expression profile post-CCI was similar to sham-conditions (Figure S4). There was no co-expression of P2X7R-EGFP with the astrocyte marker, GFAP, or the neuronal marker, synaptophysin, in sham conditions and post-CCI (Figure 2E and Figure S4), similar to observations in other seizure models 22, 50.

### P2X7R antagonism decreases TBI-induced long-lasting brain hyperexcitability, mitigates neurobehavioural deficits, and reduces cortical tissue loss

We next examined whether P2X7R antagonism reduces the observed long-lasting brain hyperexcitability post-CCI. Here, we chose to use two treatment time-windows, one starting at day 1 post-CCI (treatment window 1), and the other starting at day 7 post-CCI (treatment window 2). P2X7R antagonist treatment was administered daily for 7 days using the P2X7R antagonists JNJ-54175446 (30 mg/kg) or AFC-5128 (30 mg/kg). A separate group of mice was treated with the ASM carbamazepine (40 mg/kg) once daily following the same treatment regime as for P2X7R antagonists. Mice were then administered KA (10 mg/kg, i.p.) at day 21 post-CCI and EEG was recorded for 90 min starting at the time of KA injection to assess TBI-induced brain hyperexcitability.

**Figure 3:**
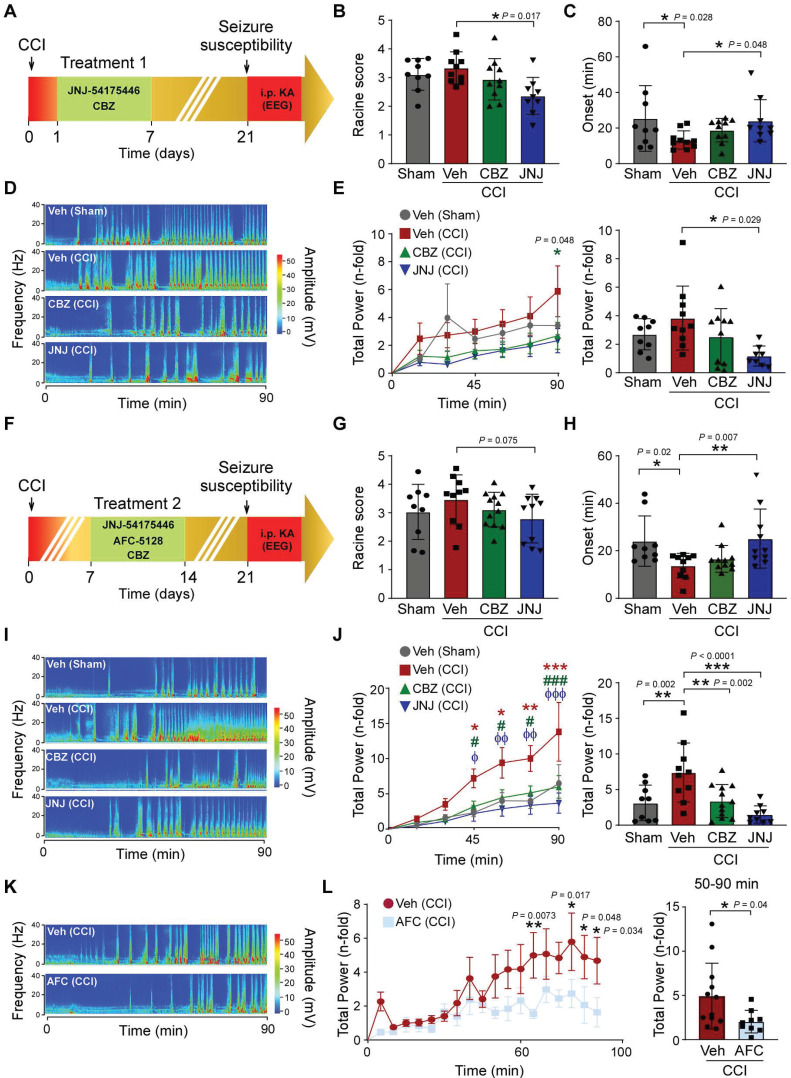
** P2X7R antagonism reduces long-lasting brain hyperexcitability post-CCI. (A)** Schematic showing experimental approach. Sham- and CCI mice were treated with the P2X7R antagonist JNJ-54175446 (30 mg/kg), carbamazepine (CBZ, 40 mg/kg) or vehicle for 7 days starting from day 1 - 7 post-sham/CCI (treatment window 1). All mice were then subjected to i.p. KA (10 mg/kg) 21 days post-sham/CCI and cortical EEG was recorded for 90 min. **(B)** Graph showing behaviour changes according to a modified Racine scale during a 90 min recording period starting from i.p. KA injection in mice treated with JNJ or vehicle (treatment window 1) (n = 9 (sham-vehicle), 10 (CCI-vehicle), 10 (CCI-CBZ) and 9 (CCI-JNJ)). One-way ANOVA followed by Fischer's multiple-comparison test. **(C)** Graph showing time to first electrographic seizure following i.p. KA (n = 9 (sham-vehicle), 10 (CCI-vehicle), 10 (CCI-CBZ) and 9 (CCI-JNJ)). One-way ANOVA followed by Fischer's multiple-comparison test. **(D)** Representative heat map and **(E)** graphs showing EEG total power over 90 min EEG recording period starting from time of i.p. KA administration in mice treated with JNJ or vehicle (treatment window 1) (left: EEG analysis of 15 min segments; right: EEG analysis of complete 90 min) (n = 9 (sham-vehicle), 10 (CCI-vehicle), 10 (CCI-CBZ) and 8 (CCI-JNJ)). One-way ANOVA followed by Fischer's multiple-comparison test. **(F)** Schematic delineating treatment window 2. Mice were treated with JNJ (30 mg/kg), AFC-5128 (30 mg/kg) or CBZ (40 mg/kg) from day 7 - 13 post-sham/CCI and administered i.p. KA at day 21 post-sham/CCI. **(G)** Graph showing behaviour changes according to a modified Racine scale during a 90 min recording period starting from time of i.p. KA injection in mice treated with JNJ, CBZ or vehicle (treatment window 2) (n = 9 (sham-vehicle), 10 (CCI-vehicle), 12 (CCI-CZB) and 10 (CCI-JNJ)). One-way ANOVA followed by Fischer's multiple-comparison test. **(H)** Graph showing time to first electrographic seizure following i.p. KA (n = 9 (sham-vehicle), 10 (CCI-vehicle), 12 (CCI-CBZ) and 10 (CCI-JNJ)). One-way ANOVA followed by Fischer's multiple-comparison test. **(I)** Representative heat maps and **(J)** graphs showing EEG total power over 90 min EEG recording period starting from time of i.p. KA administration in mice treated with JNJ, CBZ or vehicle (Veh) (treatment window 2) (left: EEG analysis of 15 min segments; right: EEG analysis of complete 90 min). Left graph: sham *vs* CCI Veh: *P = 0.02 (45 min), *P = 0.01 (60 min), **P = 0.004 (75 min), ***P = 0.0005 (90 min); CCI Veh *vs* CCI CZB: ^#^*P* = 0.04 (45 min), ^#^*P* = 0.01 (60 min), ^#^*P* = 0.01 (75 min), ^###^*P* < 0.0001 (90 min); CCI Veh *vs* CCI-JNJ: ^ϕ^*P* = 0.01 (45 min), ^ϕϕ^*P* = 0.001 (60 min), ^ϕϕ^*P* = 0.001 (75 min), ^ϕϕϕ^*P* < 0.0001 (90 min) (n = 9 (sham-vehicle), 10 (CCI-vehicle), 12 (CCI-CBZ) and 10 (CCI-JNJ)). **(K)** Representative heat map and **(L)** graphs showing EEG total power over 90 min EEG recording period starting from time of i.p. KA administration in mice subjected to CCI treated with AFC-5128 (AFC) or vehicle (treatment window 2) (n = 12 (CCI-vehicle), 9 (CCI-AFC)). Unpaired Student's t-test. We used the ROUT test (Q = 0.1%) to detect outliers in our data set. One outlier was detected and removed from Figure 3E in the JNJ-treated group. Data are presented as mean ± SD, except for Figure 3E, 3J, and 3L, where the left graph displays total power over time as mean ± SEM. *P < 0.05; **P < 0.01; ***P < 0.001.

When challenged with i.p. KA following treatment window 1 (Figure 3A), CCI mice treated with the P2X7R antagonist JNJ-54175446 experienced less severe behavioral seizures when compared to vehicle-treated CCI mice (Figure 3B). Mice treated with JNJ-54175446 also showed a longer latency to the first electrographic seizure burst (Figure 3C) and a less severe electrographic seizure phenotype during the 90 min recording period (Figure 3D-E). In contrast, carbamazepine-treated CCI mice had similar severity of seizures following KA challenge as vehicle-treated CCI animals (Figure 3B-E).

When treatment was delayed to day 7 post-CCI (treatment window 2; Figure 3F), mice subjected to CCI and treated with JNJ-54175446 had less severe behavior seizures when compared to mice subjected to CCI and treated with vehicle, although this reduction in seizures did not reach statistical significance (Figure 3G). From the EEG recordings, administration of JNJ-54175446 to CCI mice during treatment window 2 resulted in a longer latency period when compared to vehicle-treated CCI mice (Figure 3H), and JNJ-54175446-treated mice experienced less severe seizures during the 90 min recording period after i.p. KA injection (Figure 3I-J). Interestingly, carbamazepine dosing during the second treatment window also reduced seizures on the EEG when compared to vehicle-treated CCI mice (Figure 3I-J). Of note, no difference in responses to i.p. KA was observed between sham mice treated with vehicle or JNJ-54175446 (treatment window 2) 3 weeks post-injury (Figure S5). Confirming that effects are not restricted to the P2X7R antagonist JNJ-54175446, CCI-subjected mice treated with a different P2X7R antagonist, AFC-5128 (30 mg/kg, treatment window 2), also experienced less severe seizures following i.p. KA when compared to CCI-subjected mice treated with vehicle (Figure 3K-L).

**Figure 4:**
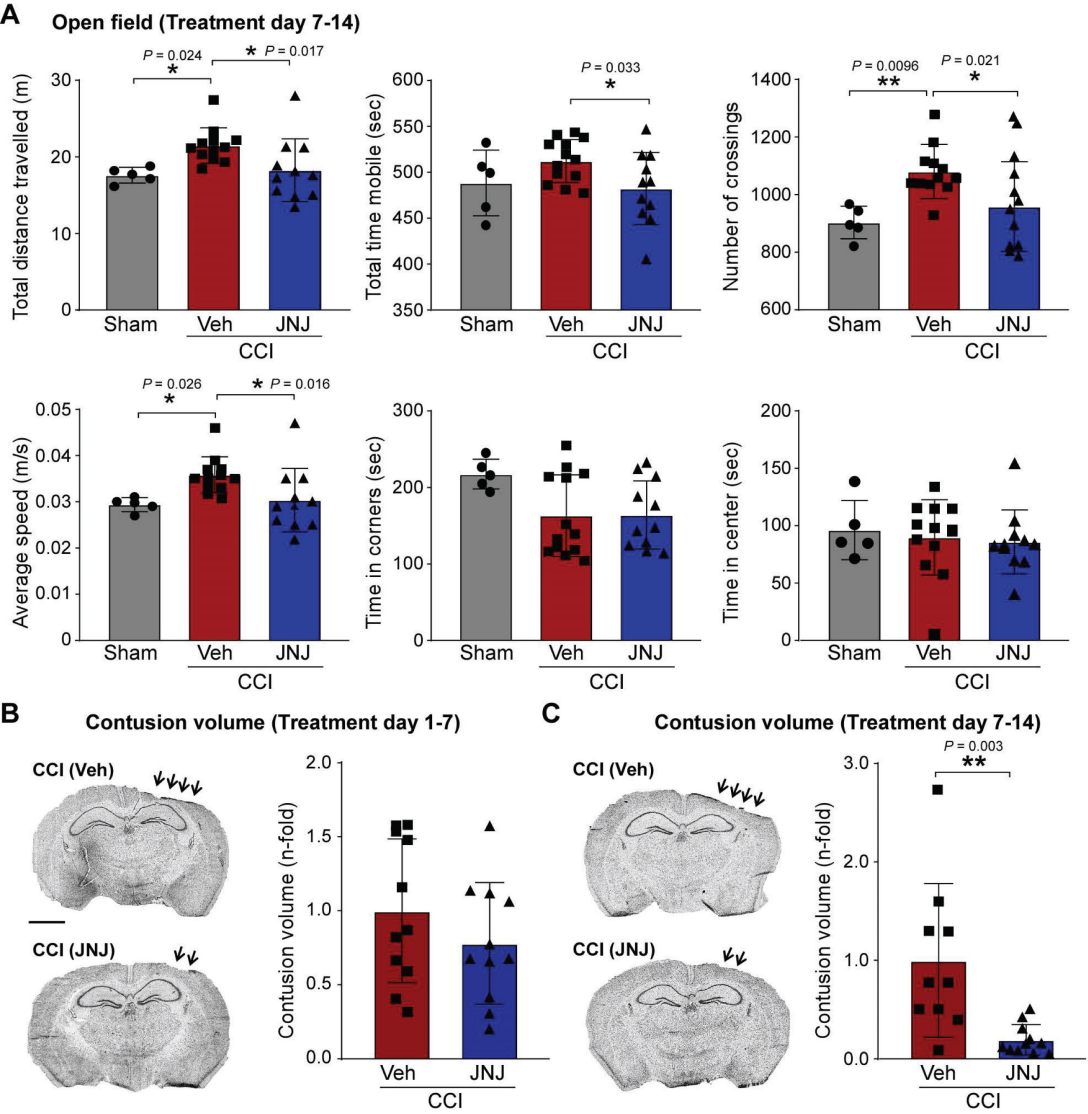
** P2X7R antagonism protects against CCI-induced tissue loss and hyperactivity. (A**) Open field analysis of mice subjected to sham and CCI treated with vehicle or P2X7R antagonist JNJ-54175446 (JNJ) during treatment window 2. Open field was carried out at day 21 post-CCI/sham. Parameters analyzed include total distance travelled, total time mobile, number of crossings, average speed, time in corners and time in center (n = 5 (sham-vehicle), 13 (CCI-vehicle) and 11 (CCI-JNJ)). One-way ANOVA followed by Fischer's multiple-comparison test. **(B-C)** Representative images and graphs showing ipsilateral cortical contusion volume in mice subjected to CCI and treated with the P2X7R antagonist JNJ-54175446 (JNJ) during treatment window 1 **(B)** (n = 11 (CCI-vehicle) and 11 (CCI-JNJ)) and treatment window 2 **(C)** (n = 10 (CCI-vehicle) and 11 (CCI-JNJ)). Scale bar = 1 mm. Unpaired Student's t-test**.** Data are shown as mean ± SD. *P < 0.05; **P < 0.01.

P2X7R antagonism has previously been shown to protect against TBI-induced neurodegeneration and behavioral deficits 30, 32. To test whether our treatments provide beneficial effects at the behavior level, mice undergoing the treatment window 2, performed an open field task at 21 days post-CCI. As expected, CCI vehicle-injected mice showed an increase in hyperactivity when compared to vehicle-treated sham mice 51 as evidenced by a longer distance travelled, more crossings and a higher average speed during a 10 min recording period (Figure 4A). Notably, in mice subjected to CCI and treated with the P2X7R antagonist JNJ-54175446, hyperactivity levels returned to sham levels (Figure 4A). No significant difference was observed between sham and CCI mice regarding time in corner and center (Figure 4A).

In our next series of experiments, we wanted to test whether P2X7R antagonism protects the brain from damage. While no significant impact on contusion volume was observed following treatment with the P2X7R antagonist JNJ-54175446 using the treatment window 1 (Figure 4B), mice subjected to CCI and treated with the P2X7R antagonist JNJ-54175446 using treatment window 2 had an approximately 60% reduction in the lesion volume when compared to vehicle-treated CCI mice (Figure 4C). This suggests that P2X7R antagonism is more effective in preventing tissue loss when P2X7R signaling is inhibited at later disease/injury stages, in line with previous studies suggesting P2X7R antagonism applied acutely following TBI can be detrimental 33.

### Microglia expressed P2X7Rs contribute to long-lasting brain hyperexcitability after TBI

To investigate whether effects of P2X7R on TBI-induced brain hyperexcitability are mediated via its effects on microglia, we analyzed brain slices from injured mice following treatment with JNJ-54175446 (or vehicle) at the end of treatment window 2 (days 7 - 13 post-injury) using the microglial marker IBA-1. While CCI led to a slight increase in IBA-1 positive microglia numbers in the ipsilateral cortex and hippocampus (Figure S6A), mice subjected to CCI and treated with the P2X7R antagonist JNJ-54175446 displayed fewer IBA-1-positive cells in the ipsilateral cortex when compared to CCI mice treated with vehicle. No difference between treatment groups was observed when analyzing the ipsilateral hippocampus (Figure S6B). To obtain further evidence of P2X7R-mediated effects on microglia contributing to TBI-induced brain hyperexcitability, mice subjected to CCI were treated with the tetracycline-type antibiotic minocycline (30 mg/kg), that in addition to its antibacterial activity also exhibits anti-inflammatory properties (*e.g*., via its effects on microglia activation 44), during the same time window as P2X7R antagonist JNJ-54175446 or vehicle treatment (treatment window 2) (Figure S6C). No difference in seizure onset and severity could be observed between groups (JNJ-54175446 vs vehicle) when treated with KA (10 mg/Kg, i.p) at day 21 post-CCI (Figure S6C), further suggesting that the effect of P2X7R antagonism on the seizure threshold post-TBI is via suppression of microglia activation.

**Figure 5:**
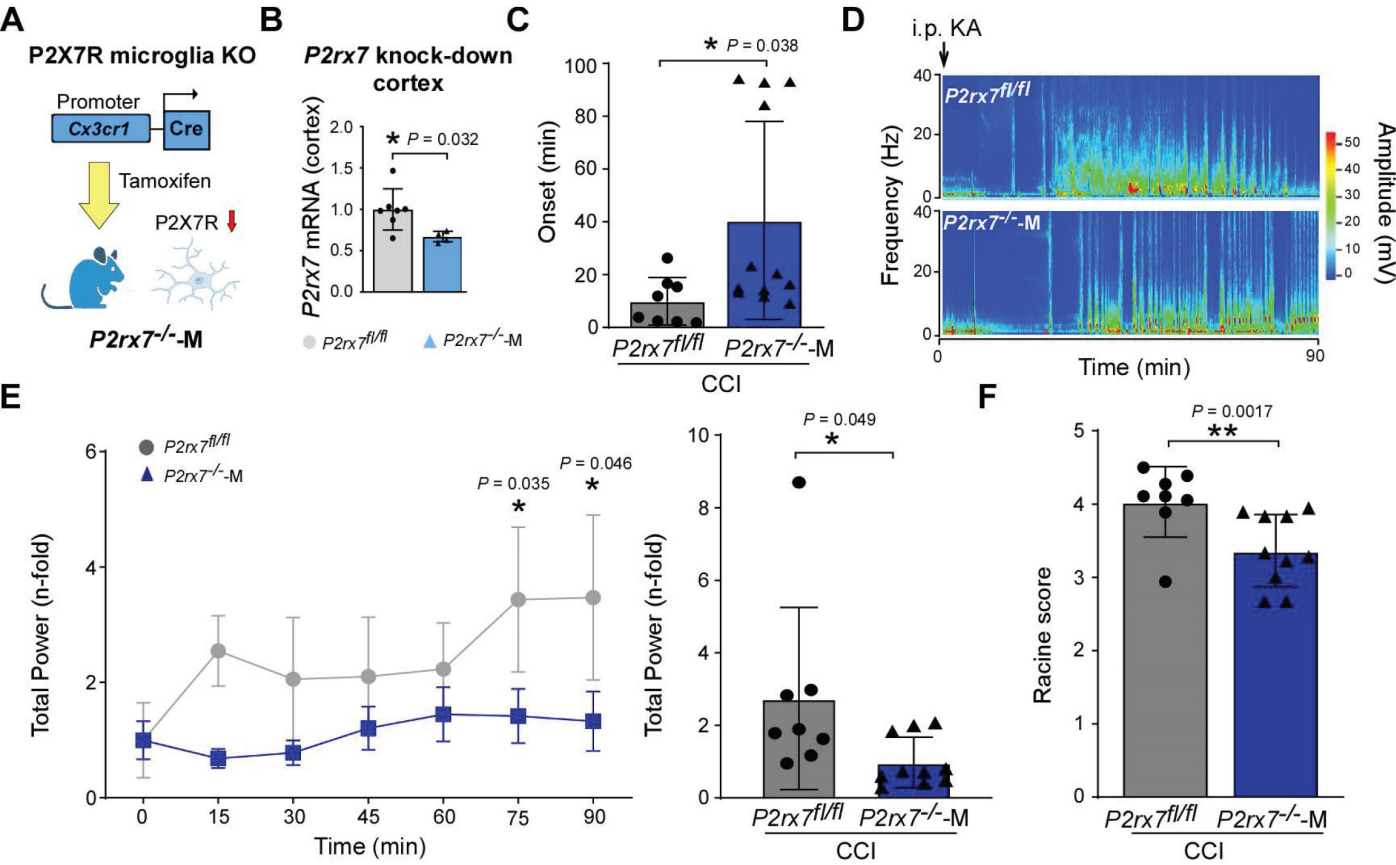
**P2X7R-mediated effects on microglia contribute to long-lasting brain hyperexcitability post-CCI. (A)** Schematic showing conditional cell type-specific knockdown of P2X7R in microglia (*P2rx7^-/-^*-M) using a tamoxifen-inducible Cre (*Cx3cr1*) line. **(B)** Cortical *P2rx7* mRNA levels post-tamoxifen treatment in naïve conditions (n = 7 (*P2rx7*^fl/fl^) and 4 (*P2rx7^-/-^*-M)). Unpaired Student's t-test. (**C**) Graphs showing seizure onset of *P2rx7^fl/fl^* and *P2rx7^-/^*^-^-M mice 21 days post-CCI subjected to i.p. KA (n = 8 (*P2rx7*^fl/fl^) and 10 (*P2rx7^-/-^*-M)). Unpaired Student's t-test. **(D)** Representative heat map of *P2rx7^fl/fl^* and *P2rx7^-/-^*-M mice 21 days post-CCI subjected to i.p. KA. **(E)** Graphs showing EEG total power during a 90 min recording period from the time of i.p. KA of *P2rx7^fl/fl^* and *P2rx7^-/^*^-^-M mice 21 days post-CCI (n = 8 (*P2rx7*^fl/fl^) and 10 (*P2rx7^-/-^*-M)) (left: EEG analysis of 15 min segments; right: EEG analysis of complete 90 min). Left graph: Two-way ANOVA. Right graph: Unpaired Student's t-test. **(F)** Graph showing behaviour changes according to a modified Racine scale during a 90 min recording period starting from i.p. KA injection in *P2rx7^fl/fl^* and *P2rx7^-/-^*-M mice 21 days post-CCI (n = 8 (*P2rx7*^fl/fl^) and 10 (*P2rx7^-/-^*-M)). Unpaired Student's t-test. Data are shown as mean ± SD. Created with BioRender.com. *P < 0.05; **P < 0.01.

Next, we generated transgenic mice lacking the *P2rx7* specifically in microglia, to obtain genetic evidence that it is P2X7Rs expressed in microglia that contributes to brain hyperexcitability after TBI. Using the conditional Cre-LoxP system, we targeted the P2X7R in microglia by crossing *P2rx7*^fl/fl^ mice with the *Cx3cr1*-Cre line, generating offspring, *P2rx7^-/-^*-M (Figure 5A). Using the same mouse model, we have previously shown *p2rx7* mRNA expression to be absent in microglia and macrophages in *P2rx7^-/-^*-M mice following tamoxifen treatment 43. In the same study we also showed that tamoxifen treatment of *P2rx7^-/-^*-M mice resulted in an approximately 25% reduction in total *p2rx7* mRNA levels in the hippocampus 43. qPCR of cortical tissue from*P2rx7^-/-^*-M mice confirmed reduced *P2rx7* mRNA levels (approximately 30%) after 7 days of tamoxifen treatment (Figure 5B).

To test whether P2X7R deletion in microglia alters the seizure threshold post-TBI, *P2rx7^-/-^*-M and control *P2rx7*^fl/fl^ mice were subjected to CCI and treated with tamoxifen for 7 consecutive days starting at 7 days post-CCI, mimicking our treatment window 2. Mice were then administered KA at 1 week after tamoxifen treatment (*i.e.,* 21 days post-CCI). This cell-specific genetic deletion experiment revealed that mice with a microglia P2X7R deletion phenocopied the results with the P2X7R antagonist, showing a longer latency towards the first electrographic seizure (Figure 5C). In addition, CCI-injured *P2rx7^-/-^*-M mice had a less severe seizure phenotype during the 90 min EEG recording period, when compared to i.p. KA-treated *P2rx7*^fl/fl^ tamoxifen-treated CCI-injured mice (Figure 5D-E). *P2rx7^-/-^*-M CCI mice also showed less severe behavioral seizures when compared *P2rx7*^fl/fl^ CCI mice in response to i.p. KA challenge (Figure 5F). While significant, we observed, however, two populations in our *P2rx7^-/-^*-M mice which may be due to an incomplete knockdown of the P2X7R in microglia or differences in underlying pathology/microglia responses. Of note, tamoxifen sham-treated *P2rx7^-/-^*-M mice showed no seizure reduction when compared to tamoxifen-treated sham *P2rx7*^fl/fl^ mice using the same tamoxifen and i.p. KA treatment regime (Figure S7). Taken together, our data suggest that the P2X7R contributes to TBI-induced hyperexcitability which is most likely mediated by its function in microglia.

### P2X7R radioligand levels following TBI correlate with seizure threshold

**Figure 6:**
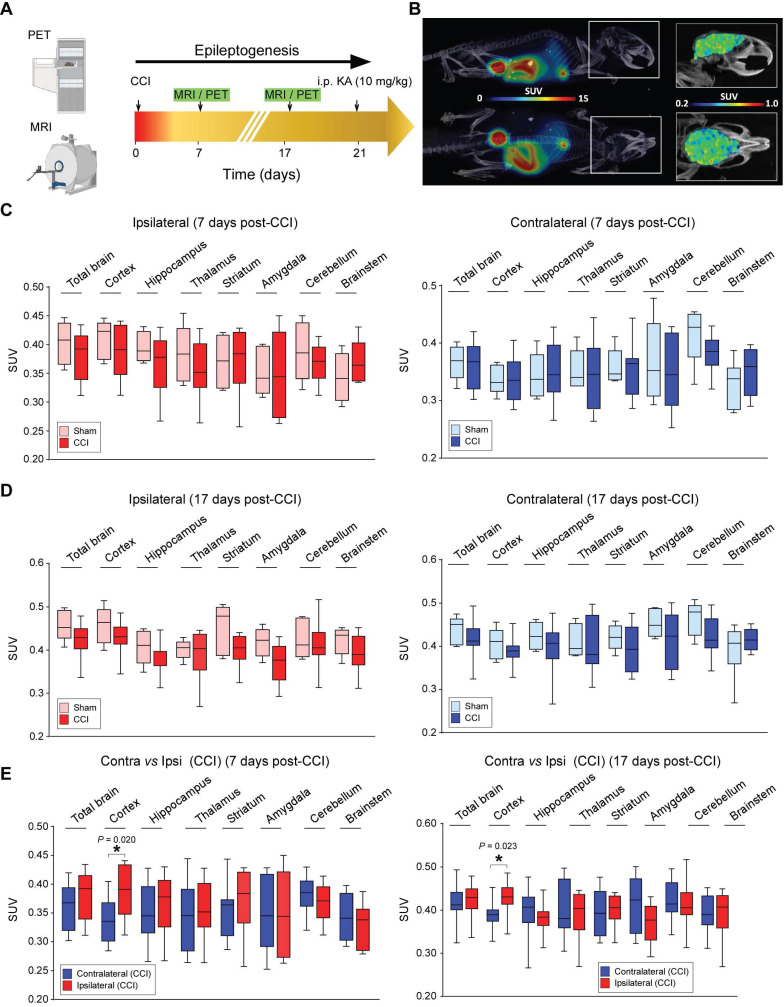
** P2X7R PET imaging following CCI. (A)** Schematic showing experimental approach. Mice were subjected to sham/CCI and MRI and PET imaging were carried out at the beginning of the 2^nd^ week post-CCI and during the 3^rd^ week post-CCI/sham. Mice where then administered KA (10 mg/kg, i.p.) and EEG recorded for 90 min. **(B)** Illustrative whole-body (left) and brain (right) PET-CT images (top: sagittal, bottom: axial) of a sham mouse used in the study. Colour scales in Standardized Uptake Values (SUV). **(C, D)** Graphs showing P2X7R radioligand uptake in ipsilateral and contralateral brain hemispheres 7 days post-sham and CCI (n = 5 (sham) and 10 (CCI), One-way ANOVA followed by Fischer's multiple-comparison test **(C)**) and 17 days post-CCI (n** =** 5 (sham) and 10 (CCI), One-way ANOVA followed by Fischer's multiple-comparison test **(D)**).** (E)** Graphs showing difference in P2X7R radioligand uptake between ipsi- and contralateral brain areas 7 days and 17 days post-CCI (n = 5 (sham) and 10 (CCI)). One-way ANOVA followed by Fischer's multiple-comparison test. Data are shown as mean ± SEM. Created with BioRender.com. *P < 0.05

The radioligand ^18^F-JNJ-64413739 has proven efficient for the determination of P2X7R expression *in vivo* 47. In a previous study performed in human subjects, kinetic modeling (1- and 2-tissue compartment models and Logan graphic analysis) using arterial blood sampling was used to estimate regional volumes of distribution, as no reference region enabling simplified quantification methods could be identified. Notably, SUV (Standardized Uptake Values) values at 30 min post-radioligand injection was previously shown to correlate with the severity of SE in mice 35. To test whether P2X7R radioligand uptake also correlates with increased brain hyperexcitability after TBI, the P2X7R radioligand ^18^F-JNJ-64413739 was injected 7 days and 17 days post-CCI (or sham) in wildtype (FVB/N) mice, and animals were subjected to KA challenge at day 21 post-CCI as before. In addition to P2X7R PET imaging, mice were also subjected to MRI at similar timepoints (Figure 6A-B).

We first compared P2X7R tracer SUVs between sham control and CCI mice. There was similar uptake of ^18^F-JNJ-64413739 when we analyzed different brain regions separately, including the ipsi- and contralateral whole brain hemisphere, cortex, hippocampus, thalamus, striatum, cerebellum, amygdala and brain stem at both the 7 and 17 days post-CCI timepoints (Figure 6C-D). P2X7R radioligand uptake was, however, higher in the ipsilateral cortex when compared to the contralateral cortex 7 days and 17 days post-CCI (Figure 6E).

Of note, similar differences between ipsilateral and contralateral brain hemispheres were observed in sham mice, suggesting that differences in P2X7R radioligand uptake may be due to the procedure itself rather than the CCI (Figure S8).

**Figure 7:**
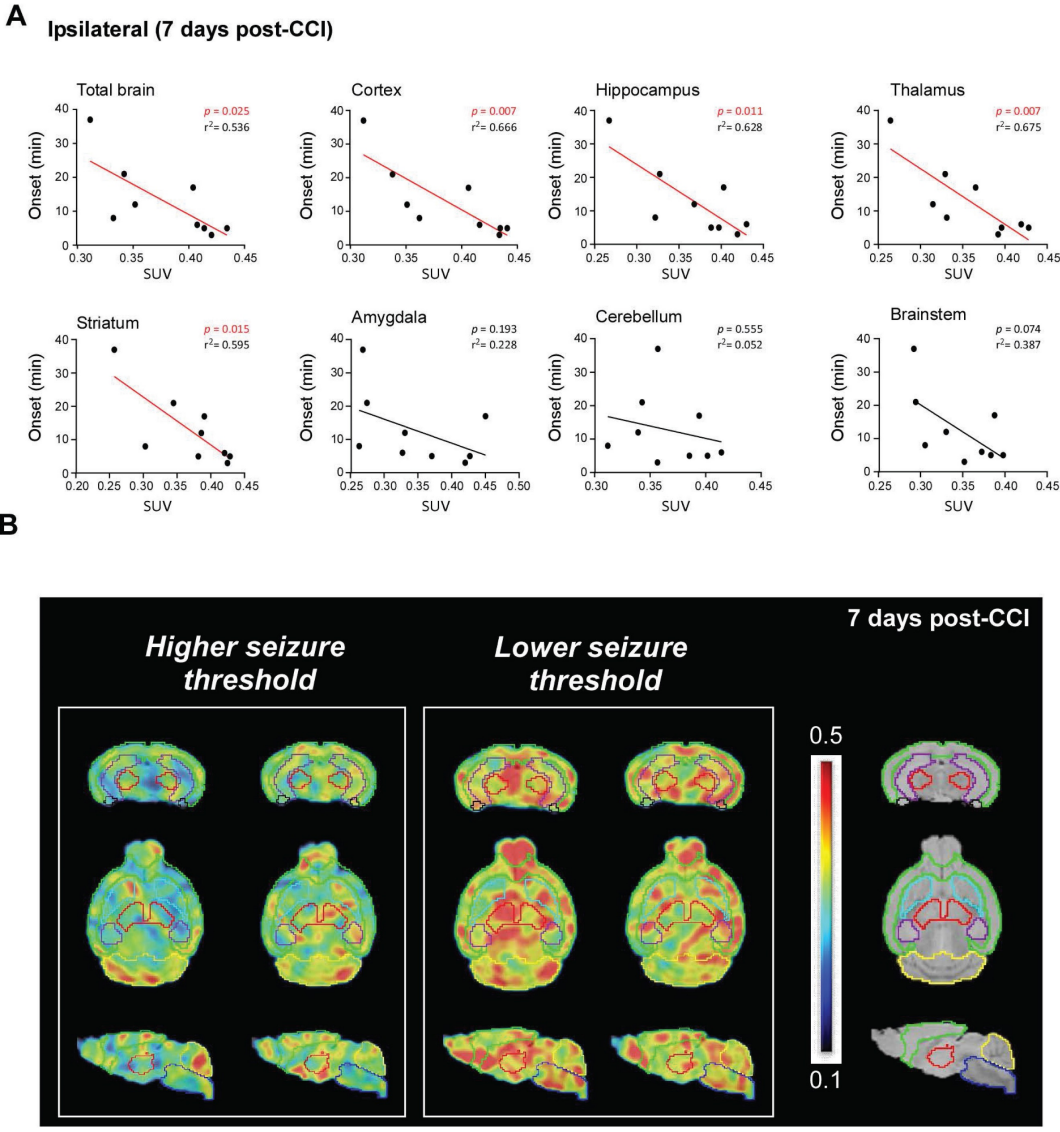
** P2X7R radioligand uptake and correlation with seizure threshold 7 days post-CCI. (A)** Graphs showing correlation between seizure onset after i.p. KA treatment (21 days post-CCI) and P2X7R radioligand uptake in different ipsilateral brain structures 7 days post-CCI (n = 9). Spearman's correlation coefficient. **(B)** Representative brain PET images (from top to bottom: coronal, axial and sagittal) of mice with a high and low seizure threshold determined via latency to first seizure post-i.p. KA treatment. The MirroneT2 MRI template is denoted on the right for regional reference (green: cortex, red: thalamus, light blue: striatum, purple: hippocampus, black: amygdala, dark blue: brain stem, yellow: cerebellum).

To determine whether P2X7R radioligand uptake changes according to susceptibility to KA at 3 weeks post-CCI, mice were subjected to the same KA challenge as before and seizure onset was recorded. There was a positive correlation between seizure onset and P2X7R radioligand uptake with mice presenting higher ^18^F-JNJ-64413739 brain uptake showing an earlier seizure onset. When analyzing the ipsilateral side, this correlation was, however, restricted to the cortex (*P* = 0.007), hippocampus (*P* = 0.011), thalamus (*P* = 0.007), and striatum (*P* = 0.015) 7 days post-CCI (Figure 7A-B). At 17 days post-CCI, this correlation was only evident in the thalamus (*P* = 0,020) and striatum (*P* = 0.010) (Figure S9). When analyzing the un-damaged contralateral side, a correlation between seizure onset and P2X7R radioligand uptake was only evident in the hippocampus (*P* = 0.023) and striatum (*P* = 0.049) at 7 days post-CCI (Table S1). Of note, no correlation between seizure onset and P2X7R radioligand brain uptake was observed when analyzing sham mice at both timepoints (Table S2). In contrast to P2X7R radioligand uptake, no obvious correlation could be observed between P2X7R PET radioligand uptake and TBI-induced cortical tissue loss quantified via MRI (Table S3A-B).

In summary, our data suggest that P2X7R-based PET with ^18^F-JNJ-64413739 may serve as a diagnostic tool to identify risk of developing increased brain hyperexcitability following TBI.

## Discussion

By using pharmacological and genetic approaches, we demonstrate that the P2X7R contributes to long-lasting brain hyperexcitability following TBI. We also show that P2X7R antagonism protects the brain from TBI-induced cortical tissue loss and neurobehavior deficits. We link these effects to P2X7R function in microglia. Finally, by using P2X7R-based PET, we show that P2X7R radioligand uptake correlates with increased long-lasting brain hyperexcitability. Our results, therefore, support the P2X7R as a promising theranostic target for PTE, with both diagnostic and therapeutic potential.

One of the major findings of our study is that P2X7R antagonism produced long-lasting seizure-suppressive effects in a mouse model of PTE. The fact that these effects extended well beyond the time of active dosing, suggests a disease-modifying potential of this approach for PTE. This is in good agreement with our previous studies showing seizure suppression in mice where epilepsy was induced via an intraamygdala injection of KA up to 7 days after drug withdrawal 27, 41, and in a mouse model of neonatal hypoxia where the hypoxia-induced pathological long-lasting brain hyperexcitability was reduced even 4 weeks following cessation of treatment with P2X7R antagonists 52.

Our data further suggest that the pro-epileptic effects of P2X7R activation appears to be mediated by microglia. Our study found a strong correlation between P2X7R expression and changes in the microglial marker IBA-1. Moreover, the seizure-suppressive effects of P2X7R antagonism were absent in the presence of minocycline, and critically, mice deficient in microglia P2X7R (*P2rx7^-/-^*-M) showed a higher seizure threshold post-TBI. Whether P2X7R alters seizure thresholds via driving neuroinflammation remains to be established. While the mechanism by which microglia promote inflammation downstream of the P2X7R during epilepsy is uncertain, it may be linked to pro-inflammatory signaling 26, 43, 53. Pro-inflammatory signaling is well established to lower the seizure threshold and inflammation is well known to be increased following TBI 54. For example, the known P2X7R target cytokine IL-1β has been shown to inhibit GABA-mediated inhibitory transmission, potentiate excitatory N-methyl-D-aspartate receptor-dependent (NMDA) synaptic transmission 55-57 and reduce the anticonvulsant action of midazolam 58. Whether P2X7R-driven inflammation contributes to the disease-modifying effects provided by P2X7R antagonism remains to be established. A possible explanation is that P2X7R antagonism breaks the vicious cycle of damage-induced increased extracellular ATP concentrations, pro-inflammatory signaling and epilepsy progression.

In addition to the seizure suppressive effects provided via P2X7R antagonism, we also observed robust P2X7R antagonism-mediated reduction of tissue loss in the cortex. These effects were, however, restricted to our second treatment window, suggesting that blocking of the P2X7R may be more beneficial during delayed brain damage following TBI rather than during acute TBI-induced injury. This is in line with a previous study suggesting that an early intervention targeting the P2X7R post-TBI is detrimental via the inhibition of the recruitment of neutrophils 33. Our results are, however, in contrast to studies by Liu *et al*. 30 and Kimbler *et al*. 31, where targeting of P2X7Rs was protective when applied before and shortly after TBI. Why P2X7R antagonism seems to be protective acutely in some studies but detrimental in others remains elusive. Possible explanations include the use of different models with different severities and/or levels of inflammatory signaling (*e.g*., CCI *vs* modified weight drop) or antagonists/methods used (*e.g*., Brilliant Blue G (BBG) *vs* JNJ-54175446 *vs* P2X7R knock-out mice). We have, however, used two different P2X7R antagonists and acute inflammatory signalling has repeatedly been shown to be protective (*e.g.,* removal of cell debris via phagocytosis 59, which has been shown to be stimulated via the P2X7R 60). Cell type-specific effects may represent another explanation, with P2X7R being active on different cell types according to disease progression.

Notably, carbamazepine, an ASM frequently used in patients with epilepsy 5, also provided protection by reducing brain hyperexcitability. However, although similar to treatment with a P2X7R antagonist, the seizure-suppressive effects of carbamazepine in our experiments were less obvious. Nevertheless, our data are in line with previous data suggesting protective effects of ASMs such as carbamazepine, phenytoin or levetiracetam which are increasingly used as a prophylactic treatment in patients to reduce the risk of PTE following TBI 61. However, contrary to currently used ASMs, which can cause serious side effects such as cognitive impairment, increased mortality, drug interactions during polytherapy, skin and hypersensitivity reactions 62, 63, P2X7R-based treatments may cause fewer adverse effects because P2X7R is believed to be activated mainly during disease conditions of high ATP release (mM range) within the pathological focus 20, thus interfering less with normal brain physiology.

The second major finding of our study points to the potential use of P2X7R radioligands as imaging biomarkers in PTE to predict long-lasting brain hyperexcitability. Specifically, we found a positive correlation between P2X7R radioligand uptake and seizure threshold, which was mostly restricted to brain areas affected by TBI, such as the ipsilateral cortex, hippocampus, or striatum. The prognostic potential of P2X7R tracer use seems, however, to decline with time, suggesting its use as an imaging biomarker of epilepsy risk would be most effective when deployed shortly following TBI. It is also important to point out that a positive correlation was only observed in approximately 60% of mice, therefore, our results should be replicated in a bigger study group in the future. Nevertheless, the fact that P2X7R antagonism suppresses long-lasting brain hyperexcitability suggests mice with higher P2X7R levels post-TBI are more prone to exhibit increased brain hyperexcitability post-TBI. Notably, we recently showed that increased P2X7R brain expression leads to a lower responsiveness to ASMs 28, thus identifying patients with higher P2X7R radioligand uptake may not only support the prognosis of PTE, but also inform on future treatment regimes.

Currently, there are no approved pharmacotherapies for TBI. Therefore, predictive blood-based and neuroimaging biomarkers are urgently needed to understand the pathobiological mechanisms that are initiated following an acute biomechanical insult to the brain that can result in delayed hyperexcitability within neural networks and chronic neurodegeneration. Our preclinical studies demonstrate that the PET imaging P2X7R radioligands can identify populations of TBI animals that will go on to develop PTE. Thus, ATP release from damaged neurons and P2X7R signaling may be key initiators of neuroinflammatory pathway activation in microglia/macrophages that results in pro-inflammatory and pro-epileptic IL-1β production that drives seizure onset and PTE development. Notably, delayed P2X7R antagonist treatment that was initiated at 7 days post-injury resulted in suppressed KA-induced seizure development and reduced cortical lesion development in TBI mice. Thus, P2X7R expression and signaling may be a pharmacodynamic biomarker of PTE development that can be monitored using novel PET imaging P2X7R radioligand. Further neuroimaging studies will be required to determine whether these promising findings can be translated to humans to predict long-lasting brain hyperexcitability in PTE patients. P2X7R antagonists are currently being developed for other neurological conditions such as depression 64, so it may be possible to repurpose safe and effective P2X7R antagonists for the treatment of PTE based on neuroimaging findings in TBI patients using P2X7R PET ligands.

P2X7Rs have been shown to be expressed in all cell types in the CNS, with particular high P2X7R expression levels in microglia 22. Whether microglial P2X7R predicts disease progression has not been addressed within this study. However, the fact that P2X7R-driven hyperexcitability seems to be mediated via microglia suggests this cell type is a promising candidate. P2X7R is, however, expressed on other cell types, such as oligodendrocytes, which may also contribute to altered P2X7R radioligand uptake. Of note, our previous study showed a strong correlation between P2X7R radioligand uptake post-SE and seizure severity during SE 35, further suggesting P2X7R PET imaging as a prognostic tool for tracking epilepsy development. Possible confounders of our PET studies include the disruption of the BBB during epilepsy 65, which may cause tracer perfusion differences that may lead to differences in tracer influx and efflux, and the presence of several loss-of-function variants identified for the P2X7R and changes in the expression for these during disease progression which may impact on P2X7R radioligand uptake 66.

While our experiments provide the proof-of-concept evidence that P2X7R signaling is involved in TBI-induced brain hyperexcitability and that blocking of the P2X7R prevents this, it is important to point out that we have used low-dose KA injections to test the effects of P2X7R antagonism on seizure thresholds. Future studies should, therefore, analyze whether P2X7R antagonism prevents or ameliorates the occurrence of epileptic seizures post-TBI. Moreover, when testing the effects of P2X7R antagonists on KA-induced seizure threshold, no significant differences were observed between the sham group and CCI mice treated with vehicle at the behaviour level, which is most likely due to variability in our control groups. Furthermore, our results suggest that the pro-convulsive effects via P2X7Rs are mediated via its action on microglia. It is, however, important to keep in mind that the anti-inflammatory actions of minocycline are not restricted to microglia and that our Cre mice do not only target microglia, but also other P2X7R-expressing cell-types including peripheral macrophages 67, 68, which have been shown to infiltrate the brain during TBI 69 and epilepsy and may contribute to changes in seizure severity 70. Moreover, our results using a P2X7R reporter mouse 22 suggest that P2X7Rs are mainly expressed in microglia and oligodendrocytes following CCI. However, we cannot exclude P2X7R expression in other cell types, such as neurons or astrocytes. In fact, a recent study has shown increased P2X7R currents in GABAergic interneurons in epilepsy in mice and increased *P2rx7* mRNA levels in neurons in TLE patients 43. Furthermore, we cannot exclude that the sham procedures may cause longer lasting injury to brain tissue (*e.g*., neuroinflammation) as previously reported 71. It is, however, important to mention that mice subjected to CCI showed a lower seizure threshold when compared to sham mice (*i.e*., 3- and 6-weeks post-injury). While outside of the scope of the present study, whether the effects of P2X7R antagonism are superior to effects of other anti-inflammatory drugs, such as minocycline 72, has not been addressed in our study. While minocycline has shown beneficial effects in several TBI models (*e.g*., 73-76, others have, however, failed to reproduce these protective effects (*e.g*., 77, 78). Finally, the present studies used only male mice, thus future studies should investigate whether P2X7R antagonism offers similar levels of protection, and via the same cell types, in female mice. Interestingly, post-traumatic microglia/macrophage activation dynamics are differentially altered following TBI with female mice displaying diminished inflammatory responses during the acute phase post-injury 79, 80, perhaps in part due to the neuroprotective actions of sex hormones 81.

## Conclusions

In summary, here we provide evidence of the antiepileptogenic potential of P2X7R antagonism and of the diagnostic potential of P2X7R-based PET for epilepsy. Importantly, P2X7R antagonists are already in clinical trials for non-epilepsy related CNS indications 64, which should accelerate development of P2X7R-based treatments for TBI-induced epilepsy and their application in the clinic in the foreseeable future.

## Supplementary Material

Supplementary materials, figures and tables.

## Abbreviations

ASM: anti-seizure medications
BBB: blood-brain barrier
CCI: controlled cortical impact
CNS: central nervous system
EEG: electroencephalogram
EGFP: enhanced green fluorescent green protein
IL-1β: interleukin-1β
i.p.: intraperitoneal
KA: kainic acid
KO: knock-out
P2X7R: P2X7 receptor
MRI: magnetic resonance imaging
PET: positron emission tomography
PTE: post-traumatic epilepsy
SE: status epilepticus
SUV: standardized uptake values
TBI: traumatic brain injury
wt: wild-type

## References

[1] Dewan MC, Rattani A, Gupta S, Baticulon RE, Hung YC, Punchak M (2018). Estimating the global incidence of traumatic brain injury. J Neurosurg.

[2] Agrawal A, Timothy J, Pandit L, Manju M (2006). Post-traumatic epilepsy: an overview. Clin Neurol Neurosurg.

[3] Ferguson PL, Smith GM, Wannamaker BB, Thurman DJ, Pickelsimer EE, Selassie AW (2010). A population-based study of risk of epilepsy after hospitalization for traumatic brain injury. Epilepsia.

[4] Bramlett HM, Dietrich WD (2015). Long-Term Consequences of Traumatic Brain Injury: Current Status of Potential Mechanisms of Injury and Neurological Outcomes. J Neurotrauma.

[5] Bialer M, White HS (2010). Key factors in the discovery and development of new antiepileptic drugs. Nat Rev Drug Discov.

[6] Thijs RD, Surges R, O'Brien TJ, Sander JW (2019). Epilepsy in adults. Lancet.

[7] Pease M, Gonzalez-Martinez J, Puccio A, Nwachuku E, Castellano JF, Okonkwo DO (2022). Risk Factors and Incidence of Epilepsy after Severe Traumatic Brain Injury. Ann Neurol.

[8] Pitkanen A, Lukasiuk K, Dudek FE, Staley KJ (2015). Epileptogenesis. Cold Spring Harb Perspect Med.

[9] Larkin M, Meyer RM, Szuflita NS, Severson MA, Levine ZT (2016). Post-Traumatic, Drug-Resistant Epilepsy and Review of Seizure Control Outcomes from Blinded, Randomized Controlled Trials of Brain Stimulation Treatments for Drug-Resistant Epilepsy. Cureus.

[10] Pitkanen A, Paananen T, Kyyriainen J, Das Gupta S, Heiskanen M, Vuokila N (2021). Biomarkers for posttraumatic epilepsy. Epilepsy Behav.

[11] Dulla CG, Pitkanen A (2021). Novel Approaches to Prevent Epileptogenesis After Traumatic Brain Injury. Neurotherapeutics.

[12] Webster KM, Sun M, Crack P, O'Brien TJ, Shultz SR, Semple BD (2017). Inflammation in epileptogenesis after traumatic brain injury. J Neuroinflammation.

[13] Kumar RG, Boles JA, Wagner AK (2015). Chronic Inflammation After Severe Traumatic Brain Injury: Characterization and Associations With Outcome at 6 and 12 Months Postinjury. J Head Trauma Rehabil.

[14] Sharma R, Leung WL, Zamani A, O'Brien TJ, Casillas Espinosa PM, Semple BD (2019). Neuroinflammation in Post-Traumatic Epilepsy: Pathophysiology and Tractable Therapeutic Targets. Brain Sci.

[15] Mukherjee S, Arisi GM, Mims K, Hollingsworth G, O'Neil K, Shapiro LA (2020). Neuroinflammatory mechanisms of post-traumatic epilepsy. J Neuroinflammation.

[16] Di Virgilio F, Sarti AC, Coutinho-Silva R (2020). Purinergic signaling, DAMPs, and inflammation. Am J Physiol Cell Physiol.

[17] Idzko M, Ferrari D, Eltzschig HK (2014). Nucleotide signalling during inflammation. Nature.

[18] Burnstock G (2016). An introduction to the roles of purinergic signalling in neurodegeneration, neuroprotection and neuroregeneration. Neuropharmacology.

[19] Beamer E, Conte G, Engel T (2019). ATP release during seizures - A critical evaluation of the evidence. Brain Res Bull.

[20] Surprenant A, Rassendren F, Kawashima E, North RA, Buell G (1996). The cytolytic P2Z receptor for extracellular ATP identified as a P2X receptor (P2X7). Science.

[21] Monif M, Reid CA, Powell KL, Smart ML, Williams DA (2009). The P2X7 receptor drives microglial activation and proliferation: a trophic role for P2X7R pore. J Neurosci.

[22] Kaczmarek-Hajek K, Zhang J, Kopp R, Grosche A, Rissiek B, Saul A (2018). Re-evaluation of neuronal P2X7 expression using novel mouse models and a P2X7-specific nanobody. Elife.

[23] Sperlagh B, Illes P (2014). P2X7 receptor: an emerging target in central nervous system diseases. Trends Pharmacol Sci.

[24] Engel T (2023). The P2X7 Receptor as a Mechanistic Biomarker for Epilepsy. Int J Mol Sci.

[25] Engel T, Smith J, Alves M (2021). Targeting Neuroinflammation via Purinergic P2 Receptors for Disease Modification in Drug-Refractory Epilepsy. J Inflamm Res.

[26] Engel T, Gomez-Villafuertes R, Tanaka K, Mesuret G, Sanz-Rodriguez A, Garcia-Huerta P (2012). Seizure suppression and neuroprotection by targeting the purinergic P2X7 receptor during status epilepticus in mice. FASEB J.

[27] Jimenez-Pacheco A, Diaz-Hernandez M, Arribas-Blazquez M, Sanz-Rodriguez A, Olivos-Ore LA, Artalejo AR (2016). Transient P2X7 Receptor Antagonism Produces Lasting Reductions in Spontaneous Seizures and Gliosis in Experimental Temporal Lobe Epilepsy. J Neurosci.

[28] Beamer E, Morgan J, Alves M, Menendez Mendez A, Morris G, Zimmer B (2022). Increased expression of the ATP-gated P2X7 receptor reduces responsiveness to anti-convulsants during status epilepticus in mice. Br J Pharmacol.

[29] Wang YC, Cui Y, Cui JZ, Sun LQ, Cui CM, Zhang HA (2015). Neuroprotective effects of brilliant blue G on the brain following traumatic brain injury in rats. Mol Med Rep.

[30] Liu X, Zhao Z, Ji R, Zhu J, Sui QQ, Knight GE (2017). Inhibition of P2X7 receptors improves outcomes after traumatic brain injury in rats. Purinergic Signal.

[31] Kimbler DE, Shields J, Yanasak N, Vender JR, Dhandapani KM (2012). Activation of P2X7 promotes cerebral edema and neurological injury after traumatic brain injury in mice. PLoS One.

[32] Kobayashi M, Moro N, Yoshino A, Kumagawa T, Shijo K, Maeda T (2023). Inhibition of P2X4 and P2X7 receptors improves histological and behavioral outcomes after experimental traumatic brain injury in rats. Exp Ther Med.

[33] Roth TL, Nayak D, Atanasijevic T, Koretsky AP, Latour LL, McGavern DB (2014). Transcranial amelioration of inflammation and cell death after brain injury. Nature.

[34] Fu Z, Lin Q, Xu Z, Fu W, Shi D, Cheng Y (2022). Longitudinal Positron Emission Tomography Imaging with P2X7 Receptor-Specific Radioligand (18)F-FTTM in a Kainic Acid Rat Model of Temporal Lobe Epilepsy. ACS Chem Neurosci.

[35] Morgan J, Moreno O, Alves M, Baz Z, Menendez Mendez A, Leister H (2023). Increased uptake of the P2X7 receptor radiotracer (18) F-JNJ-64413739 in the brain and peripheral organs according to the severity of status epilepticus in male mice. Epilepsia.

[36] Krieg SM, Sonanini S, Plesnila N, Trabold R (2015). Effect of small molecule vasopressin V1a and V2 receptor antagonists on brain edema formation and secondary brain damage following traumatic brain injury in mice. J Neurotrauma.

[37] Alves M, De Diego Garcia L, Conte G, Jimenez-Mateos EM, D'Orsi B, Sanz-Rodriguez A (2019). Context-Specific Switch from Anti- to Pro-epileptogenic Function of the P2Y1 Receptor in Experimental Epilepsy. J Neurosci.

[38] Jimenez-Mateos EM, Engel T, Merino-Serrais P, McKiernan RC, Tanaka K, Mouri G (2012). Silencing microRNA-134 produces neuroprotective and prolonged seizure-suppressive effects. Nat Med.

[39] Loane DJ, Kumar A, Stoica BA, Cabatbat R, Faden AI (2014). Progressive neurodegeneration after experimental brain trauma: association with chronic microglial activation. J Neuropathol Exp Neurol.

[40] Fischer W, Franke H, Krugel U, Muller H, Dinkel K, Lord B (2016). Critical Evaluation of P2X7 Receptor Antagonists in Selected Seizure Models. PLoS One.

[41] Mamad O, Heiland M, Lindner AU, Hill TDM, Ronroy RM, Rentrup K (2023). Anti-seizure effects of JNJ-54175446 in the intra-amygdala kainic acid model of drug-resistant temporal lobe epilepsy in mice. Front Pharmacol.

[42] Hammond FM, Zafonte RD, Tang Q, Jang JH (2021). Carbamazepine for Irritability and Aggression after Traumatic Brain Injury: A Randomized, Placebo-Controlled Study. J Neurotrauma.

[43] Alves M, Gil B, Villegas-Salmeron J, Salari V, Martins-Ferreira R, Arribas Blazquez M (2024). Opposing effects of the purinergic P2X7 receptor on seizures in neurons and microglia in male mice. Brain Behav Immun.

[44] Panizzutti B, Skvarc D, Lin S, Croce S, Meehan A, Bortolasci CC (2023). Minocycline as Treatment for Psychiatric and Neurological Conditions: A Systematic Review and Meta-Analysis. Int J Mol Sci.

[45] Engel T, Sanz-Rodgriguez A, Jimenez-Mateos EM, Concannon CG, Jimenez-Pacheco A, Moran C (2013). CHOP regulates the p53-MDM2 axis and is required for neuronal survival after seizures. Brain.

[46] Wehn AC, Khalin I, Duering M, Hellal F, Culmsee C, Vandenabeele P (2021). RIPK1 or RIPK3 deletion prevents progressive neuronal cell death and improves memory function after traumatic brain injury. Acta Neuropathol Commun.

[47] Koole M, Schmidt ME, Hijzen A, Ravenstijn P, Vandermeulen C, Van Weehaeghe D (2019). (18)F-JNJ-64413739, a Novel PET Ligand for the P2X7 Ion Channel: Radiation Dosimetry, Kinetic Modeling, Test-Retest Variability, and Occupancy of the P2X7 Antagonist JNJ-54175446. J Nucl Med.

[48] Di Sapia R, Moro F, Montanarella M, Iori V, Micotti E, Tolomeo D (2021). In-depth characterization of a mouse model of post-traumatic epilepsy for biomarker and drug discovery. Acta Neuropathol Commun.

[49] Schneider M, Prudic K, Pippel A, Klapperstuck M, Braam U, Muller CE (2017). Interaction of Purinergic P2X4 and P2X7 Receptor Subunits. Front Pharmacol.

[50] Morgan J, Alves M, Conte G, Menendez-Mendez A, de Diego-Garcia L, de Leo G (2020). Characterization of the Expression of the ATP-Gated P2X7 Receptor Following Status Epilepticus and during Epilepsy Using a P2X7-EGFP Reporter Mouse. Neurosci Bull.

[51] Popovitz J, Mysore SP, Adwanikar H (2019). Long-Term Effects of Traumatic Brain Injury on Anxiety-Like Behaviors in Mice: Behavioral and Neural Correlates. Front Behav Neurosci.

[52] Smith J, Menendez Mendez A, Alves M, Parras A, Conte G, Bhattacharya A (2023). The P2X7 receptor contributes to seizures and inflammation-driven long-lasting brain hyperexcitability following hypoxia in neonatal mice. Br J Pharmacol.

[53] Giuliani AL, Sarti AC, Falzoni S, Di Virgilio F (2017). The P2X7 Receptor-Interleukin-1 Liaison. Front Pharmacol.

[54] Sun L, Shan W, Yang H, Liu R, Wu J, Wang Q (2021). The Role of Neuroinflammation in Post-traumatic Epilepsy. Front Neurol.

[55] Wang S, Cheng Q, Malik S, Yang J (2000). Interleukin-1beta inhibits gamma-aminobutyric acid type A (GABA(A)) receptor current in cultured hippocampal neurons. J Pharmacol Exp Ther.

[56] Yang S, Liu ZW, Wen L, Qiao HF, Zhou WX, Zhang YX (2005). Interleukin-1beta enhances NMDA receptor-mediated current but inhibits excitatory synaptic transmission. Brain Res.

[57] Roseti C, van Vliet EA, Cifelli P, Ruffolo G, Baayen JC, Di Castro MA (2015). GABAA currents are decreased by IL-1beta in epileptogenic tissue of patients with temporal lobe epilepsy: implications for ictogenesis. Neurobiol Dis.

[58] Clarkson BDS, Kahoud RJ, McCarthy CB, Howe CL (2017). Inflammatory cytokine-induced changes in neural network activity measured by waveform analysis of high-content calcium imaging in murine cortical neurons. Sci Rep.

[59] Sochocka M, Diniz BS, Leszek J (2017). Inflammatory Response in the CNS: Friend or Foe?. Mol Neurobiol.

[60] Gu BJ, Wiley JS (2018). P2X7 as a scavenger receptor for innate phagocytosis in the brain. Br J Pharmacol.

[61] Wat R, Mammi M, Paredes J, Haines J, Alasmari M, Liew A (2019). The Effectiveness of Antiepileptic Medications as Prophylaxis of Early Seizure in Patients with Traumatic Brain Injury Compared with Placebo or No Treatment: A Systematic Review and Meta-Analysis. World Neurosurg.

[62] Fordington S, Manford M (2020). A review of seizures and epilepsy following traumatic brain injury. J Neurol.

[63] Huo X, Xu X, Li M, Xiao L, Wang Y, Li W (2022). Effectiveness of antiseizure medications therapy in preventing seizures in brain injury patients: A network meta-analysis. Front Pharmacol.

[64] Recourt K, de Boer P, van der Ark P, Benes H, van Gerven JMA, Ceusters M (2023). Characterization of the central nervous system penetrant and selective purine P2X7 receptor antagonist JNJ-54175446 in patients with major depressive disorder. Transl Psychiatry.

[65] Marchi N, Granata T, Ghosh C, Janigro D (2012). Blood-brain barrier dysfunction and epilepsy: pathophysiologic role and therapeutic approaches. Epilepsia.

[66] Fuller SJ, Stokes L, Skarratt KK, Gu BJ, Wiley JS (2009). Genetics of the P2X7 receptor and human disease. Purinergic Signal.

[67] Zhao XF, Alam MM, Liao Y, Huang T, Mathur R, Zhu X (2019). Targeting Microglia Using Cx3cr1-Cre Lines: Revisiting the Specificity. eNeuro.

[68] Ren W, Rubini P, Tang Y, Engel T, Illes P (2021). Inherent P2X7 Receptors Regulate Macrophage Functions during Inflammatory Diseases. Int J Mol Sci.

[69] Kumar A, Alvarez-Croda DM, Stoica BA, Faden AI, Loane DJ (2016). Microglial/Macrophage Polarization Dynamics following Traumatic Brain Injury. J Neurotrauma.

[70] Bosco DB, Tian DS, Wu LJ (2020). Neuroimmune interaction in seizures and epilepsy: focusing on monocyte infiltration. FEBS J.

[71] Lagraoui M, Latoche JR, Cartwright NG, Sukumar G, Dalgard CL, Schaefer BC (2012). Controlled cortical impact and craniotomy induce strikingly similar profiles of inflammatory gene expression, but with distinct kinetics. Front Neurol.

[72] Bergold PJ, Furhang R, Lawless S (2023). Treating Traumatic Brain Injury with Minocycline. Neurotherapeutics.

[73] Sanchez Mejia RO, Ona VO, Li M, Friedlander RM (2001). Minocycline reduces traumatic brain injury-mediated caspase-1 activation, tissue damage, and neurological dysfunction. Neurosurgery.

[74] Sangobowale MA, Grin'kina NM, Whitney K, Nikulina E, St Laurent-Ariot K, Ho JS (2018). Minocycline plus N-Acetylcysteine Reduce Behavioral Deficits and Improve Histology with a Clinically Useful Time Window. J Neurotrauma.

[75] Celorrio M, Shumilov K, Payne C, Vadivelu S, Friess SH (2022). Acute minocycline administration reduces brain injury and improves long-term functional outcomes after delayed hypoxemia following traumatic brain injury. Acta Neuropathol Commun.

[76] Lu Q, Xiong J, Yuan Y, Ruan Z, Zhang Y, Chai B (2022). Minocycline improves the functional recovery after traumatic brain injury via inhibition of aquaporin-4. Int J Biol Sci.

[77] Pechacek KM, Reck AM, Frankot MA, Vonder Haar C (2022). Minocycline fails to treat chronic traumatic brain injury-induced impulsivity and attention deficits. Exp Neurol.

[78] Scott G, Zetterberg H, Jolly A, Cole JH, De Simoni S, Jenkins PO (2018). Minocycline reduces chronic microglial activation after brain trauma but increases neurodegeneration. Brain.

[79] Villapol S, Loane DJ, Burns MP (2017). Sexual dimorphism in the inflammatory response to traumatic brain injury. Glia.

[80] Doran SJ, Ritzel RM, Glaser EP, Henry RJ, Faden AI, Loane DJ (2019). Sex Differences in Acute Neuroinflammation after Experimental Traumatic Brain Injury Are Mediated by Infiltrating Myeloid Cells. J Neurotrauma.

[81] Gupte R, Brooks W, Vukas R, Pierce J, Harris J (2019). Sex Differences in Traumatic Brain Injury: What We Know and What We Should Know. J Neurotrauma.

