# Supplementary material for: P2X7R antagonism suppresses long-lasting brain hyperexcitability following traumatic brain injury in mice
Source: Theranostics. 2025 Jan 27;15(4):1399–419. doi: 10.7150/thno.97254 (PMC11780721; doi:10.7150/thno.97254)
Supplement: Supplementary file 1 — Supplementary materials, figures and tables. [file thnov15p1399s1.pdf]

Supplementary Material

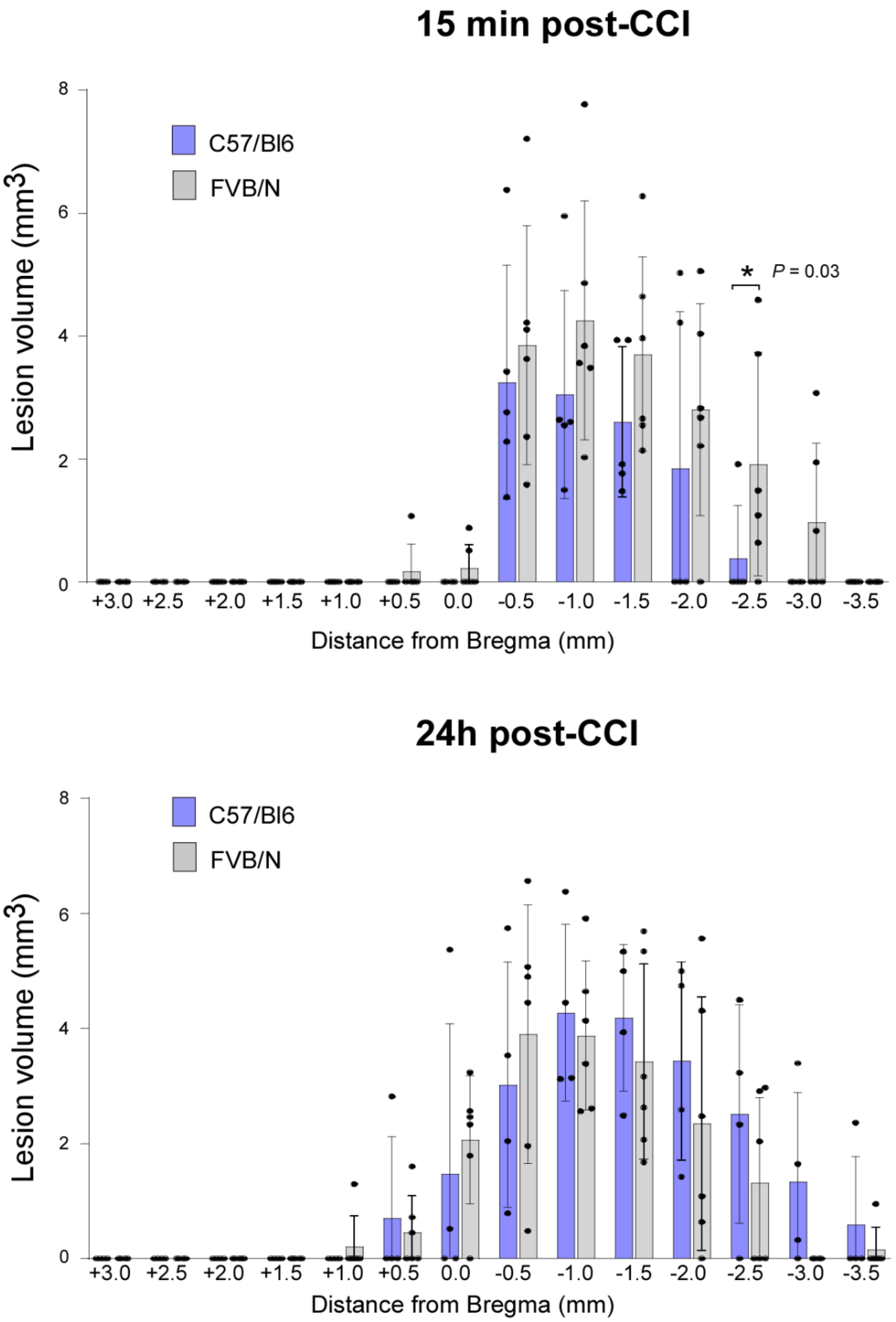

Figure S1: Contusion volume comparison between different mouse strains. (A) Analysis of the lesion volume of FBV/N mouse strain compared to C57Bl/6 mice at 15 min **(A)** and 24 h **(B)** post-CCI (15 min: n = 5 C57/Bl6 and 6 FVB/N; 24 h: n = 4 C57/Bl6 and 6 FVB/N). Data are shown as mean ± SD. **P* < 0.05.

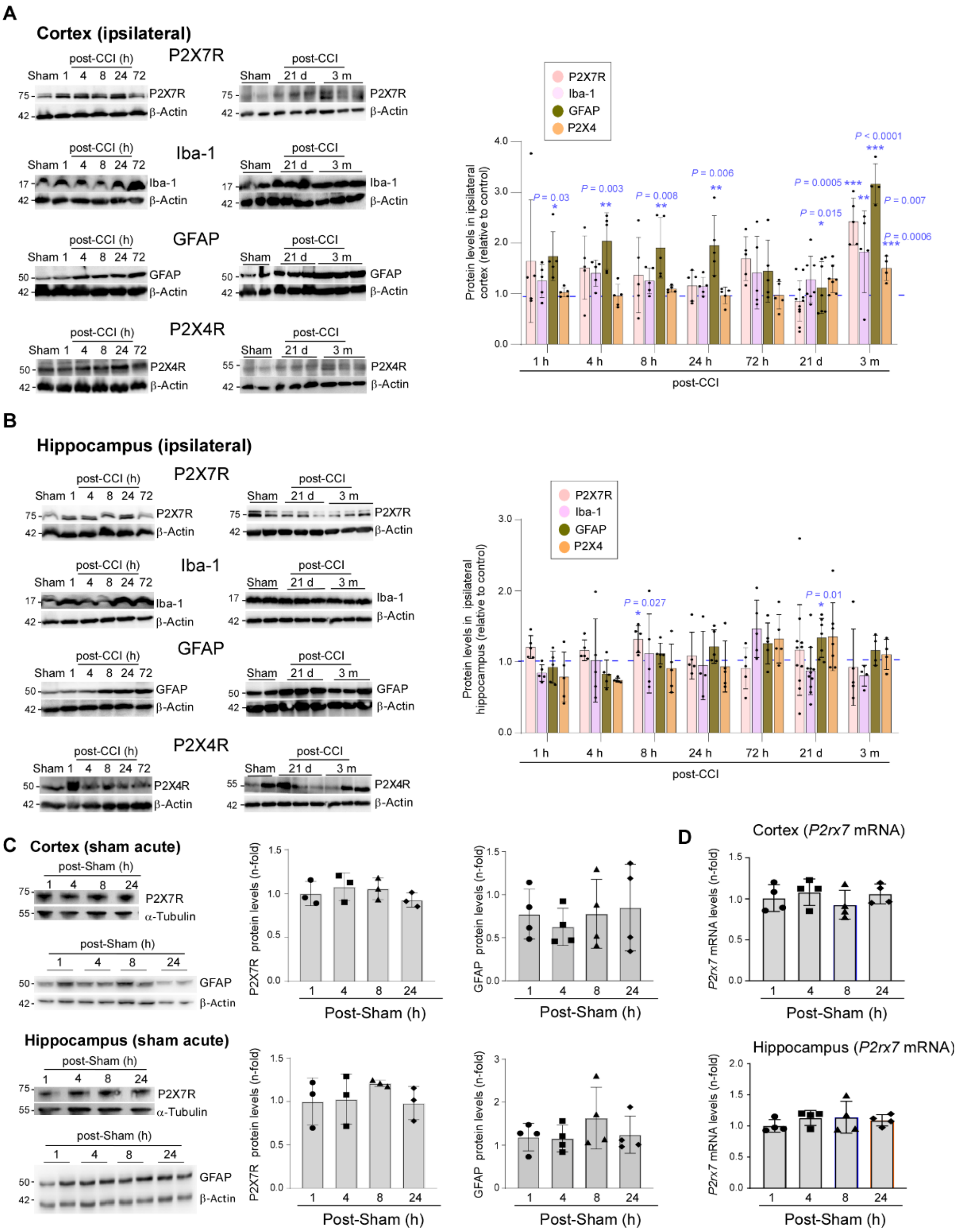

Figure S2: Expression changes of P2X7R, P2X4R and astrocyte and microglia-specific genes post-CCI. (A) Representative Western blots and corresponding graphs showing expression of the microglia-specific gene IBA-1 and astrocyte gene GFAP, P2X7R (Alomone Labs) and P2X4R post-CCI in the cortex (n = 5 (24 h and 72 h sham: P2X7R, Iba-1, GFAP, P2X4R); 5 (post-CCI: 1 h, 4 h, 8 h, 24 h and 72 h: P2X7R, Iba-1, GFAP, P2X4R); 15 (sham: 21 days and 3 months: P2X7R, IBA-1); 8 (sham: 21 days and 3 months: GFAP, P2X4R); 11 (post-CCI: 21 days: P2X7R); 7 (post-CCI: 21 days: IBA-1, P2X4R, GFAP); 5 (post-CCI: 3 months:P2X7R, IBA-1) and 4 (post-CCI: 3 months: P2X4R, GFAP). **(B)** Representative Western blots and corresponding graphs showing expression of the microglia-specific gene IBA-1 and astrocyte gene GFAP, P2X7R and P2X4R post-CCI in the hippocampus (n = 5 (24 h and 72 h sham: P2X7R, Iba-1, GFAP, P2X4R); 5 (post-CCI: 1 h, 4 h, 8 h, and 72 h: P2X7R, Iba-1, GFAP, P2X4R); 5 (post-CCI: 24 h: P2X7R, P2X4R, GFAP); 4 (post-CCI: 24 h: Iba-1); 15 (sham: 21 days and 3 months: P2X7R, IBA-1); 8 (sham: 21 days and 3 months combined: GFAP, P2X4R); 11 (post-CCI: 21 days: P2X7R and Iba-1); 7 (post-CCI: 21 days: P2X4R and GFAP);

5 (post-CCI: 3 months: P2X7R); 4 (post-CCI: 3months: P2X4R, Iba-1, GFAP). One-way ANOVA followed by Fischer's multiple-comparison test. **(C)** Western blot and graphs showing P2X7R and GFAP protein levels in the hippocampus and cortex 1 h, 4 h, 8 h and 24 h after sham-injury (n = 3 (P2X7) and 4 (GFAP) per group). **(D)** *P2rx7* mRNA levels in the hippocampus and cortex after sham (n = 4 per group). Data are shown as mean ± SD. $*P < 0.05$; $**P < 0.01$; $***P < 0.001$.

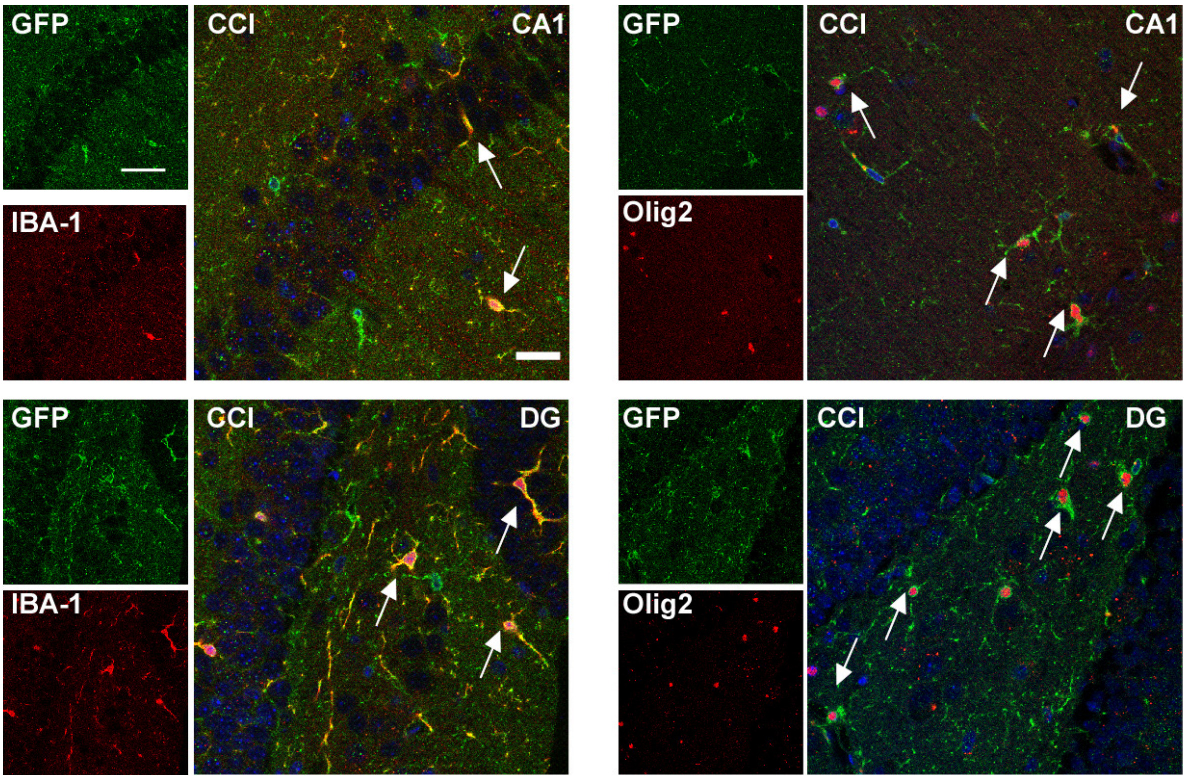

Figure S3: Co-expression of P2X7-GFP with cell type-specific markers post-CCI. Representative images showing co-expression of GFP (green) with IBA-1 (red) and Olig2 (red) 21 days post-CCI in the CA1 and hilus of the hippocampus. DAPI is shown in blue. Scale bar = 100 µm (small images, left) and 20 µm (merge image, right).

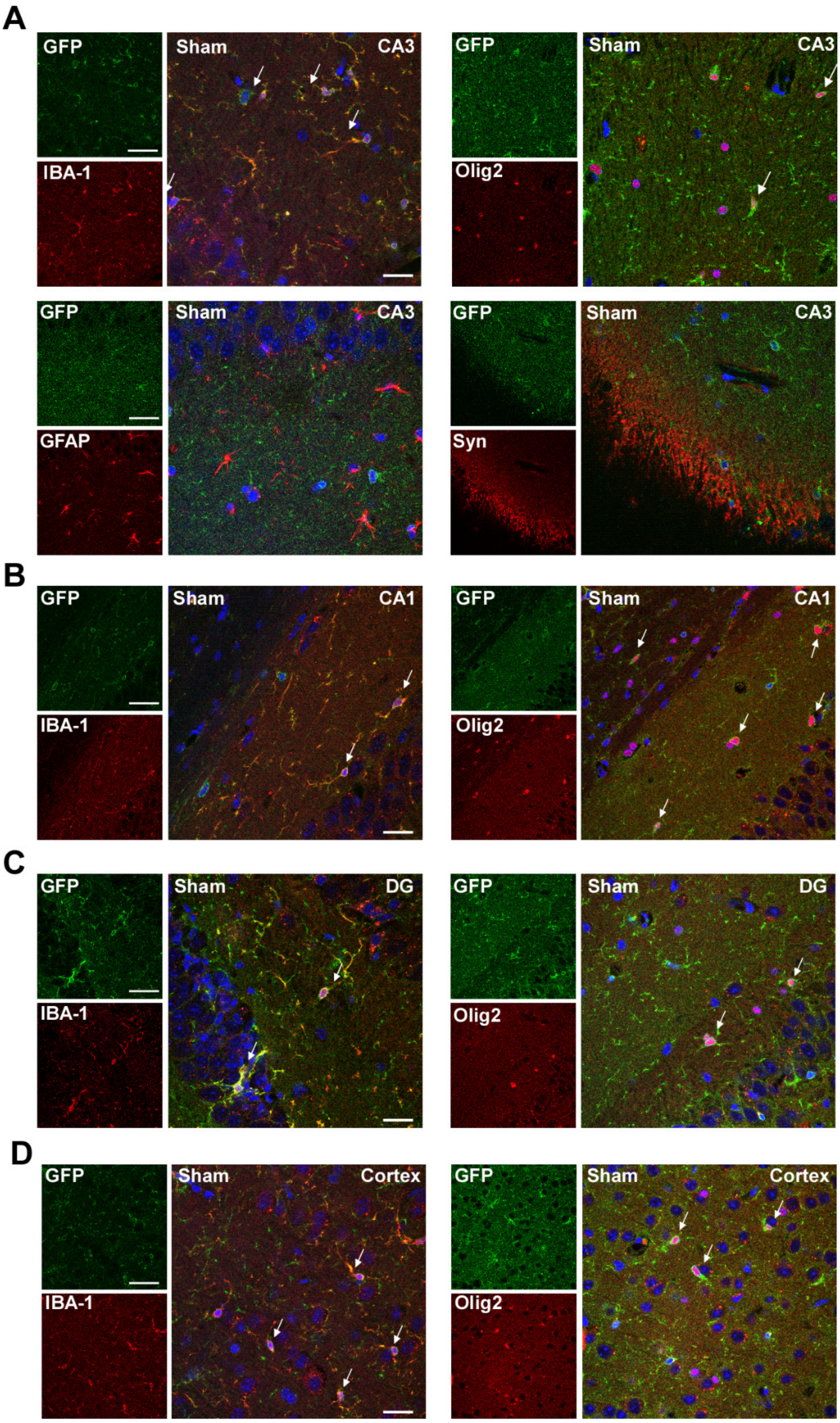

Figure S4: Co-expression of P2X7-GFP with cell type-specific markers post-sham. (A) Representative images showing co-expression of GFP (green) with IBA-1 (red) and Olig2 (red) and synaptophysin 21 days post-sham in the hippocampal subfield CA3. Representative images showing co-expression of IBA-1 and Olig2 with GFP in CA1 **(B)**, DG **(C)** and cortex **(D)**. DAPI is shown in blue. Scale bar = 100 µm (small images, left) and 20 µm (merge image, right).

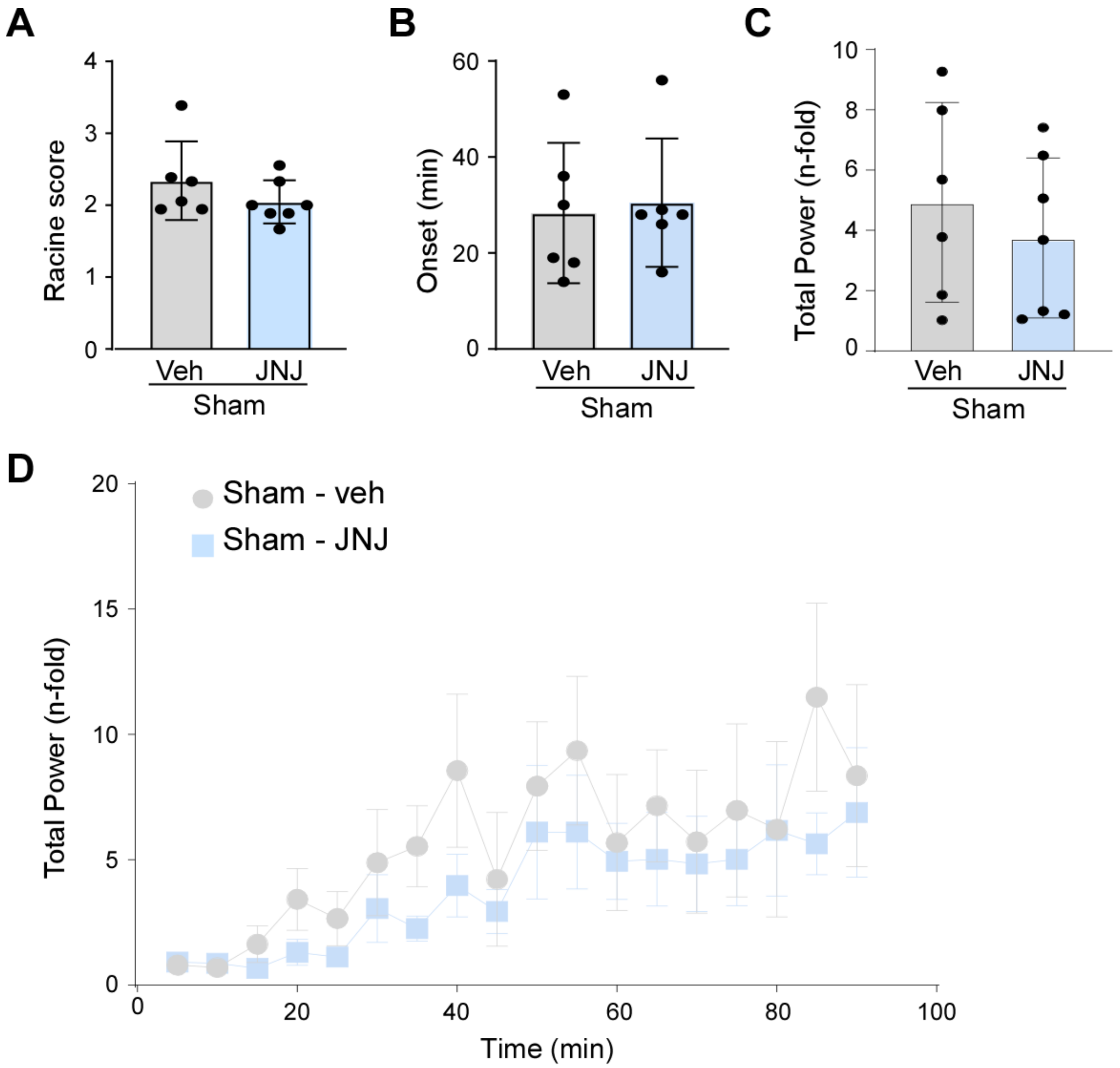

Figure S5: Seizure threshold in JNJ-treated mice 21 days post-sham using treatment window 2. **(A)** Graph showing behaviour changes according to a modified Racine scale 21 days post-sham during a 90 min recording period starting from i.p. KA injection in mice treated with JNJ or vehicle (treatment window 2) (n = 6 (sham-vehicle) and 7 (sham-JNJ)). Unpaired Student's t-test. **(B)** Graph showing time to first electrographic seizure following i.p. KA 21 days post-injury (n = 6 (sham-vehicle) and 7 (sham-JNJ; one sham mouse was excluded as seizure activity of individual seizures was below 15 s). Unpaired Student's t-test. **(C)** Graph showing EEG total power over 90 min EEG recording period starting from time of i.p. KA administration in mice treated with JNJ or vehicle (n = 6 (sham-vehicle) and 7 (sham-JNJ)). Unpaired Student's t-test. **(D)** Total power analysis starting at the time of i.p. KA injection for 90 min. EEG analysis was divided into 15 min segments (n = 6 (sham-vehicle) and 7 (sham-JNJ). One-way ANOVA followed by Fischer's multiple-comparison test. Data are presented as mean ± SD, except for (**5D**), where the graph displays total power over time as mean ± SEM.

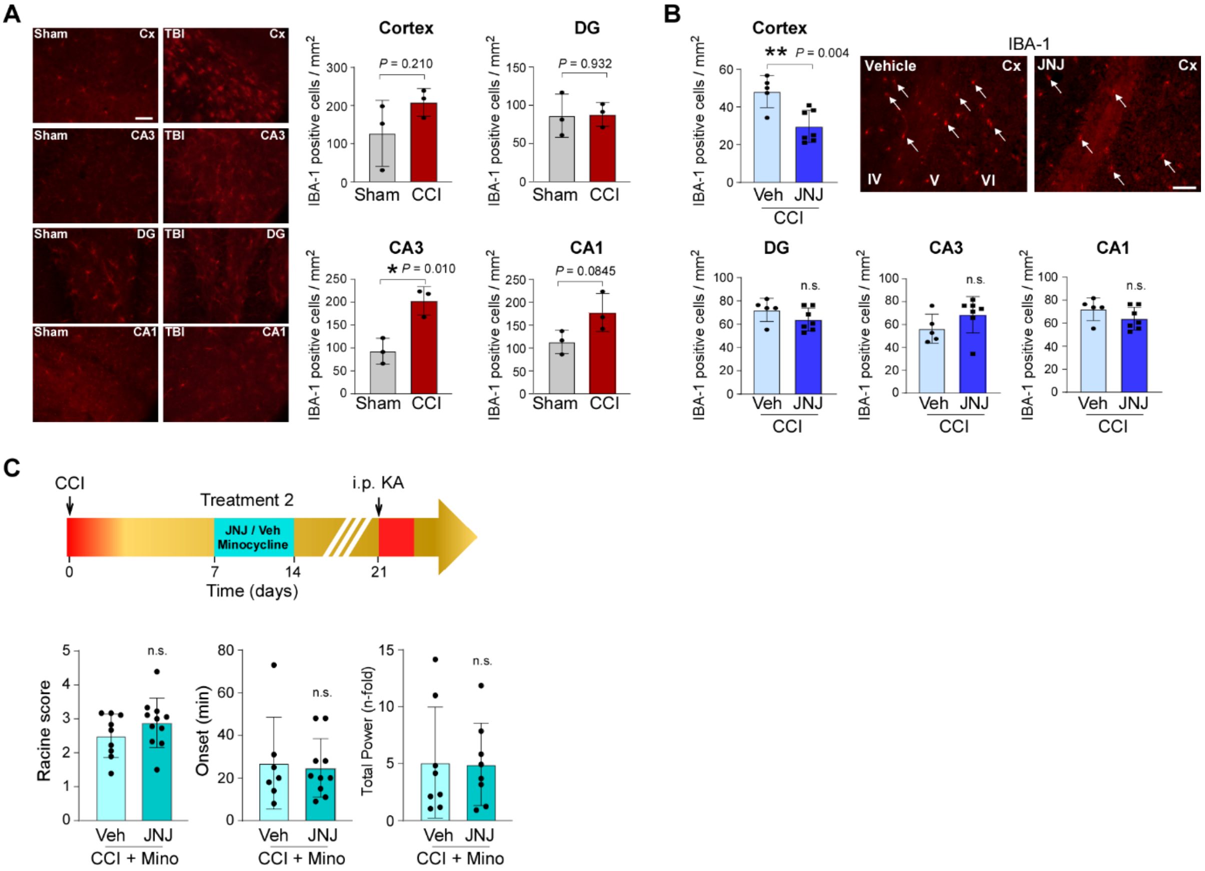

Figure S6: Effects of P2X7R signalling on microglia post-CCI. **(A)** Representative images and corresponding graphs showing IBA-1 positive cells in the ipsilateral cortex and hippocampal subfields in mice 21 days post-sham and CCI (n = 3 per group, brain slices were 30 µm thick). Scale bar = 50 µm. Unpaired Student's t-test. **(B)** Representative images and corresponding graphs showing IBA-1 positive cells in the ipsilateral cortex and hippocampal subfields in mice 21 days post-CCI. Mice were treated with the P2X7R antagonist JNJ-54175446 (JNJ) or vehicle as before using treatment window 2 (n = 5 (CCI-vehicle) and 7 (CCI-JNJ), brain slices were 12 µm thick). Scale bar = 50 µm. Unpaired Student's t-test. **(C)** Experimental design using the broad-spectrum inflammation blocker minocycline (Mino). Mice subjected to CCI were treated with minocycline (30 mg/kg, i.p) at the same time-points as treated with the P2X7R antagonist JNJ (treatment window 2, 7 - 13 days post-CCI) or vehicle and injected with KA (10 mg/kg, i.p.). Graphs showing similar seizure onset, behaviour seizures and total EEG power during a 90 min recording period starting at the time of i.p. KA administration between mice subjected to CCI and treated with minocycline and vehicle when compared to mice subjected to CCI and treated with minocycline and JNJ. Unpaired Student's t-test. *$P < 0.05$, **$P < 0.01$; n.s: no significant

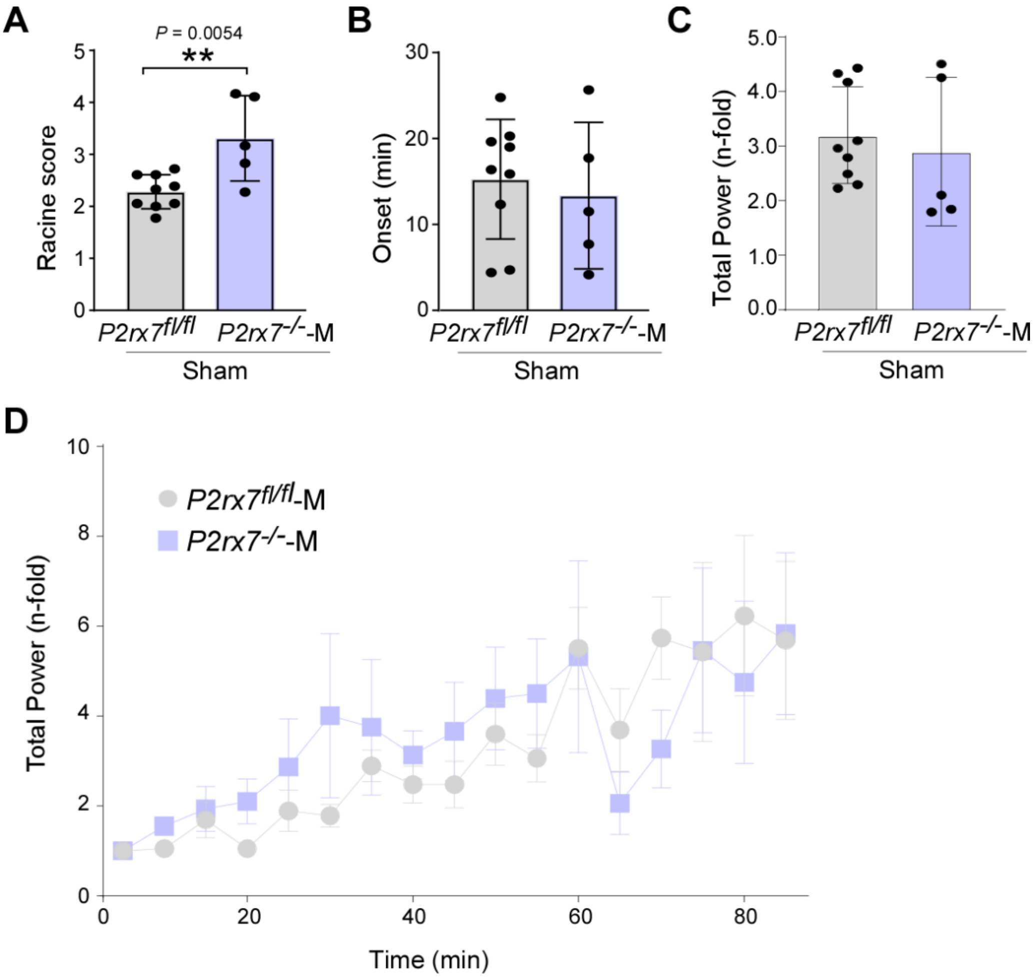

Figure S7: Seizure threshold in *P2rx7$^{-/-}$*-M mice 21 days post-sham. **(A)** Graph showing behaviour changes according to a modified Racine scale 21 days post-sham during a 90 min recording period starting from i.p. KA injection in tamoxifen-treated (treatment window 2) *P2rx7*$^{fl/fl}$ and *P2rx7$^{-/-}$-M* mice (n = 9 (*P2rx7*$^{fl/fl}$) and 5 (*P2rx7$^{-/-}$-M*)). Unpaired Student's t-test. **(B)** Graph showing time to first electrographic seizure following i.p. KA 21 days post-injury (n = 9 (*P2rx7*$^{fl/fl}$) and 5 (*P2rx7$^{-/-}$-M*)). Unpaired Student's t-test. **(C)** Graphs showing EEG total power over 90 min EEG recording period starting from time of i.p. KA administration in tamoxifen-treated (treatment window 2) *P2rx7*$^{fl/fl}$ and *P2rx7$^{-/-}$-M* mice (n = 9 (*P2rx7*$^{fl/fl}$) and 5 (*P2rx7$^{-/-}$-M*)). Unpaired Student's t-test. **(D)** Total power analysis starting at the time of i.p. KA injection for 90 min. EEG analysis was divided into 15 min segments (n = *P2rx7*$^{fl/fl}$ and *P2rx7$^{-/-}$-M* mice). One-way ANOVA followed by Fischer's multiple-comparison test. Data are presented as mean ± SD, except for **7D**, where the graph displays total power over time as mean ± SEM. ***$P < 0.01$

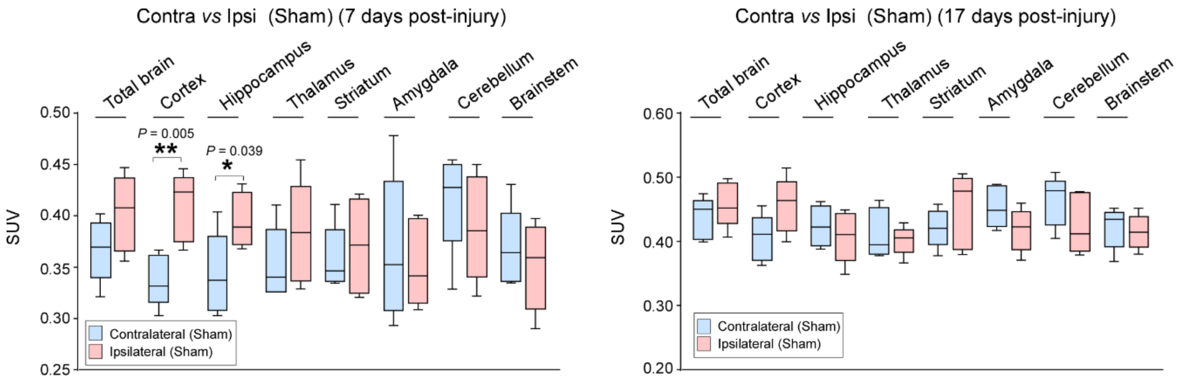

Figure S8: P2X7R radioligand uptake in sham mice. Graphs showing difference between ipsi- and contralateral P2X7R radioligand uptake 7 days and 17 days post-Sham (n = 5 per group). One-way ANOVA followed by Fischer's multiple-comparison test. Data are shown as mean ± SEM. *$P < 0.05$; **$P < 0.01$.

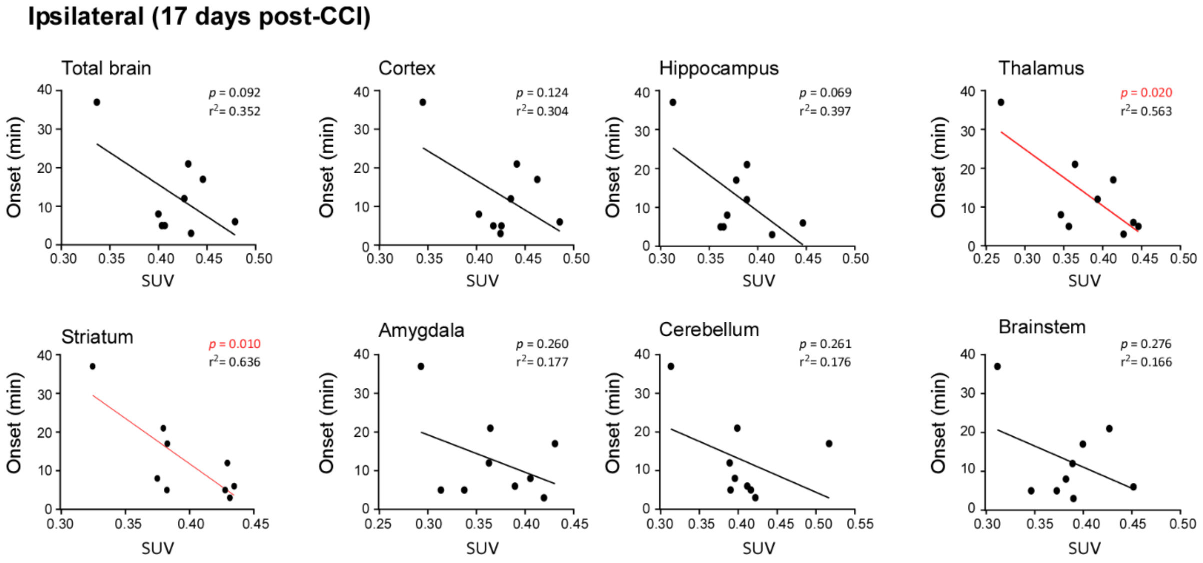

Figure S9: P2X7R radioligand uptake 17 days-post-CCI and correlation with seizure threshold 21 days post-CCI. Graphs showing correlation between seizure onset after i.p. KA treatment (21 days post-CCI) and P2X7R radioligand uptake in different ipsilateral brain structures 17 days post-CCI (n = 9). Spearman's correlation coefficient.

Supplementary Tables

Table S1: *Correlation of P2X7R radioligand uptake in contralateral brain hemisphere and seizure onset post-KA, when P2X7R PET imaging was carried out 7- and 17-days post-CCI.*

	**7 days post-CCI (n = 10)**	**17 days post-CCI (n = 10)**
Whole Brain	r^2 =0.404; p = 0.066	r^2 = 0.352; p = 0.092
Cortex	r^2 = 0.322; p = 0.111	r^2 = 0.329; p = 0.106
Hippocampus	r^2 = 0.545; p = 0.023	r^2 = 0.435; p = 0.053
Striatum	r^2 = 0.446; p = 0.049	r^2 = 0.264; p = 0.15
Thalamus	r^2 = 0.302; p = 0.125	r^2 = 0.060; p = 0.527
Amygdala	r^2 = 0.347; p = 0.095	r^2 = 0.380; p = 0.077
Brain stem	r^2 = 0.412; p = 0.063	r^2 = 0.137; p = 0.540
Cerebellum	r^2 = 0.051; p = 0.557	r^2 = 0.163; p = 0.284

Table S2: *Correlation of P2X7R radioligand uptake and seizure onset post-KA when P2X7R PET imaging was carried out 7- and 17-days post-injury (sham).*

		7 days post-Sham (n = 5)	17 days post-Sham (n = 5)
Whole Brain	ipsilateral	$r^2 = 0.235$; $p = 0.408$	$r^2 = 0.137$; $p = 0.540$
	contralateral	$r^2 = 0.496$; $p = 0.14$	$r^2 = 0.285$; $p = 0.354$
Cortex	ipsilateral	$r^2 = 0.046$; $p = 0.703$	$r^2 = 0.422$; $p = 0.235$
	contralateral	$r^2 = 0.521$; $p = 0.169$	$r^2 = 0.457$; $p = 0.210$
Hippocampus	ipsilateral	$r^2 = 0.281$; $p = 0.358$	$r^2 = 0.222$; $p = 0.423$
	contralateral	$r^2 = 0.309$; $p = 0.330$	$r^2 = 0.279$; $p = 0.360$
Striatum	ipsilateral	$r^2 = 0.329$; $p = 0.31$	$r^2 = 0.082$; $p = 0.640$
	contralateral	$r^2 = 0.073$; $p = 0.661$	$r^2 = 0.091$; $p = 0.622$
Thalamus	ipsilateral	$r^2 = 0.388$; $p = 0.262$	$r^2 = 0.034$; $p = 0.765$
	contralateral	$r^2 = 0.566$; $p = 0.142$	$r^2 = 0.505$; $p = 0.178$
Amygdala	ipsilateral	$r^2 = 0.387$; $p = 0.262$	$r^2 = 0.089$; $p = 0.626$
	contralateral	$r^2 = 0.459$; $p = 0.20$	$r^2 = 0.158$; $p = 0.507$
Brain stem	ipsilateral	$r^2 = 0.052$; $p = 0.712$	$r^2 = 0.009$; $p = 0.881$
	contralateral	$r^2 = 0.622$; $p = 0.113$	$r^2 = 0.321$; $p = 0.320$
Cerebellum	ipsilateral	$r^2 = 0.113$; $p = 0.58$	$r^2 = 0.404$; $p = 0.249$
	contralateral	$r^2 = 0.384$; $p = 0.265$	$r^2 = 0.179$; $p = 0.478$

Table S3A: *Correlation of P2X7R radioligand uptake and cortical brain damage measured at same time-points post-CCI (diagnostic).*

		**1st imaging post-CCI (PET and MRI) (n = 10)**	**2nd imaging post-CCI (PET and MRI) (n = 10)**
Whole Brain	ipsilateral	$r^2 = 0.217$; $p = 0.175$	$r^2 = 0.331$; $p = 0.082$
	contralateral	$r^2 = 0.109$; $p = 0.352$	$r^2 = 0.194$; $p = 0.203$
Cortex	ipsilateral	$r^2 = 0.121$; $p = 0.324$	$r^2 = 0.361$; $p = 0.066$
	contralateral	$r^2 = 0.025$; $p = 0.663$	$r^2 = 0.092$; $p = 0.391$
Hippocampus	ipsilateral	$r^2 = 0.047$; $p = 0.548$	$r^2 = 0.301$; $p = 0.101$
	contralateral	$r^2 = 0.009$; $p = 0.780$	$r^2 = 0.146$; $p = 0.276$
Striatum	ipsilateral	$r^2 = 0.090$; $p = 0.789$	$r^2 = 0.162$; $p = 0.249$
	contralateral	$r^2 = 0.018$; $p = 0.708$	$r^2 = 0.027$; $p = 0.651$
Thalamus	ipsilateral	$r^2 = 0.046$; $p = 0.383$	$r^2 = 0.333$; $p = 0.081$
	contralateral	$r^2 = 0.196$; $p = 0.221$	$r^2 = 0.289$; $p = 0.109$
Amygdala	ipsilateral	$r^2 = 0.441$; $p = 0.036$	$r^2 = 0.181$; $p = 0.220$
	contralateral	$r^2 = 0.120$; $p = 0.326$	$r^2 = 0.001$; $p = 0.925$
Brain stem	ipsilateral	$r^2 = 0.374$; $p = 0.060$	$r^2 = 0.299$; $p = 0.102$
	contralateral	$r^2 = 0.193$; $p = 0.205$	$r^2 = 0.547$; $p = 0.015$
Cerebellum	ipsilateral	$r^2 = 0.339$; $p = 0.076$	$r^2 = 0.162$; $p = 0.249$
	contralateral	$r^2 = 0.393$; $p = 0.052$	$r^2 = 0.060$; $p = 0.496$

Table S3B: *Correlation of P2X7R radioligand uptake at 7 days and cortical brain damage assessed during 2nd imaging post-CCI (prognostic).*

		**PET at 7 days post-CCI and MRI at 16-21 days post-CCI (n = 10)**
Whole Brain	ipsilateral	$r^2 = 0.107$; $p = 0.356$
	contralateral	$r^2 = 0.179$; $p = 0.224$
Cortex	ipsilateral	$r^2 = 0.051$; $p = 0.529$
	contralateral	$r^2 = 0.255$; $p = 0.136$
Hippocampus	ipsilateral	$r^2 = 0.125$; $p = 0.316$
	contralateral	$r^2 = 0.234$; $p = 0.156$
Striatum	ipsilateral	$r^2 = 0.239$; $p = 0.152$
	contralateral	$r^2 = 0.071$; $p = 0.456$
Thalamus	ipsilateral	$r^2 = 0.223$; $p = 0.169$
	contralateral	$r^2 = 0.247$; $p = 0.144$
Amygdala	ipsilateral	$r^2 = 0.032$; $p = 0.618$
	contralateral	$r^2 = 0.204$; $p = 0.190$
Brain stem	ipsilateral	$r^2 = 0.030$; $p = 0.631$
	contralateral	$r^2 = 0.144$; $p = 0.280$
Cerebellum	ipsilateral	$r^2 = 0.032$; $p = 0.621$
	contralateral	$r^2 = 0.000$; $p = 0.970$

Supplementary Material for experimental details

Radiochemistry

^{18}F-JNJ-64413739 synthesis was performed using a TRACERlab FX_{FN} synthesis module (GE Healthcare) following a previously described method with minor modifications [35]. $[^{18}F]F^{-}$ was generated in a Cyclone 18/9 cyclotron (IBA, Belgium) by proton irradiation of ^{18}O-enriched water via the $^{18}O(p,n)^{18}F$ nuclear reaction, and trapped on a preconditioned Sep-Pak Accell Plus QMA Light cartridge (Waters). The trapped $[^{18}F]F^{-}$ was eluted with a solution of Kryptofix $K_{2.2.2}/K_2CO_3$ in a mixture of acetonitrile/water (2:1, 1.5 ml). After complete elimination of the solvent by azeotropic evaporation, a solution containing the precursor (JNJ-64410047, 4.0 mg) in dimethylsulfoxide (0.7 ml) was added, and the mixture was heated at 120°C for 15 min. After cooling to room temperature, the mixture was purified by high-performance liquid chromatography (HPLC) using a Phenomenex Luna C18 (250 mm) column as the stationary phase and water (0.1% trifluoroacetic acid/acetonitrile (65/35, vol/vol) as the mobile phase (flow rate = 4 ml/min). The desired fraction (retention time = 18.0 min) was collected, diluted with a sodium ascorbate solution, and reformulated using a C-18 light cartridge (Sep-Pak Plus, Waters). The resulting ethanol solution (1 ml) was diluted with 9 ml of sodium ascorbate solution to yield an injectable solution of the radiotracer. Chemical and radiochemical purity were determined by radio-HPLC, and identity of the desired tracer was confirmed by coelution with reference standard. An Agilent Eclipse XBD-C18 (4.6 × 150 mm, 5 μm) was used as the stationary phase and water (0.1% trifluoroacetic acid)/acetonitrile (70/30, vol/vol) as the mobile phase at a flow rate of 1 ml/min (retention time = 12.5 min). Decay-corrected radiochemical yield at the end of the synthesis was 6.9 ± .5% (total synthesis time: 90 min). Radiochemical purity was >99%, and molar activity values were within the range of 25–95 GBq/μ mol at the end of the synthesis (n = 4).
